# Optimization and Characterization of a Physicochemical Decellularization Protocol for Porcine Tracheal Scaffolds

**DOI:** 10.3390/bioengineering13101181

**Published:** 2026-10-10

**Authors:** Elizabete Silva dos Santos, Ronaldo Soares da Silva, Sabrina da Silva de Morais, Fabíola Matos da Cruz, Hector Fugihara Kroes, Marina Maizato, Idagene Aparecida Cestari, Paulo Manuel Pêgo-Fernandes, Aristides Tadeu Correia, Paulo Francisco Guerreiro Cardoso

**Affiliations:** 1 Programa de Pos-Graduacao em Cirurgia Toracica e Cardiovascular, Faculdade de Medicina FMUSP, Universidade de Sao Paulo, Sao Paulo 01246-903, SP, Brazil; 2 Laboratorio de Pesquisa em Cirurgia Toracica, Departamento de Cardiopneumologia, Instituto do Coracao (InCor), Hospital das Clinicas HCFMUSP, Universidade de Sao Paulo, Sao Paulo 05403-000, SP, Brazilpaulo.cardoso@hc.fm.usp.br (P.F.G.C.); 3 Bioengenharia, Instituto do Coracao do Hospital das Clinicas HCFMUSP, Faculdade de Medicina FMUSP, Universidade de Sao Paulo, Sao Paulo 05403-000, SP, Brazil

**Keywords:** trachea, tissue engineering, extracellular matrix, biocompatible materials, tracheal stenosis, bioengineering, regenerative medicine

## Abstract

Reconstruction of extensive tracheal defects remains a challenge, and the development of decellularized scaffolds represents a promising strategy for tissue engineering. This study aimed to optimize a porcine tracheal decellularization protocol using sodium deoxycholate (SDC), reducing detergent exposure time without compromising extracellular matrix (ECM) preservation or biomechanical properties. Ten decellularization cycles were evaluated by residual DNA quantification, histological analysis, and residual SDC quantification, and cycles 1, 2, and 10 were selected for ECM characterization, cytocompatibility assessment, and biomechanical analysis. The first cycle was effective for submucosal decellularization, although chondrocytes remained within the tracheal cartilage. ECM architecture and its biomechanical properties were preserved. Additional cycles provided no further benefit to decellularization and were associated with alterations in longitudinal biomechanical parameters. Residual SDC was detected in the scaffolds, and undiluted extracts showed cell viability below 70%. These results indicate that 24 h of SDC exposure achieved effective submucosal decellularization while chondrocytes remained in the cartilage regardless of the number of decellularization cycles. ECM characteristics and tissue biomechanical properties were preserved.

## 1. Introduction

Reconstruction of extensive tracheal defects remains a challenge in thoracic surgery because of the structural and functional complexity of the trachea and the limitations of currently available substitutes [1]. Its specialized tissue organization provides mechanical strength and flexibility, which are essential for maintaining airway patency. In this context, tissue engineering and the development of decellularized extracellular matrices represent promising strategies for tracheal reconstruction. Preservation of extracellular matrix (ECM) components and architecture may provide a favorable microenvironment for cell adhesion, migration, proliferation, and differentiation, making these matrices potential scaffolds for regenerative medicine applications [2,3,4,5].

Decellularization aims to remove cellular and nuclear components while preserving, as much as possible, ECM structure and composition. Its efficiency depends on the methods employed and on parameters such as reagent concentration, temperature, agitation, and exposure time. However, cell removal must be balanced against ECM preservation, because excessively aggressive conditions may alter structural proteins, glycosaminoglycans, bioactive factors, and tissue biomechanical properties [2,5,6]. Sodium deoxycholate (SDC) is used in decellularization protocols for different tissues, including the trachea, and can promote efficient removal of cellular components while preserving aspects of ECM architecture [6,7,8]. However, prolonged exposure to detergents may promote matrix alterations and compromise structural and biomechanical characteristics [6].

Tracheal decellularization is challenging because of its heterogeneous composition and the structural organization of cartilage. The complete chondrocyte removal that lies within the cartilage demands a longer exposure to the decellularizing agent, that potentially increases the ECM damage. The complete removal of chondrocytes may not be necessary given their low immunogenic potential compared to the epithelium, glandular tissue, and perichondrium. Experimental studies demonstrated absent or low expression of molecules related to antigen presentation in tracheal chondrocytes and no significant rejection response after transplantation of matrices containing residual chondrocytes [9,10,11,12].

These findings suggest that decellularization may be directed primarily toward removing cellular components with greater immunogenic potential, avoiding prolonged detergent exposure aimed exclusively at chondrocyte removal and thereby favoring ECM preservation.

In this context, decellularization efficiency should not be determined exclusively by cell removal, but rather by comprehensive characterization of the resulting scaffold. Residual DNA is an important assessment parameter, whereas analysis of ECM components provides information on matrix preservation and biomechanical evaluation determines whether properties compatible with native tissue function are maintained [2,3,6,13]. In addition, residual detergents may remain associated with the tissue and interfere with cell viability, making quantification of these residues and assessment of cytocompatibility relevant to the characterization of decellularized scaffolds.

The literature on tracheal decellularization shows considerable heterogeneity regarding the agents used, concentrations, exposure times, and washing strategies [5]. Recent experimental studies have explored different chemical and processing strategies for tracheal decellularization, including combinations of decellularizing agents and partial decellularization approaches, highlighting the importance of balancing effective cell removal with preservation of ECM components and biomechanical properties [14,15]. Despite advances, there remains a need for protocols that combine efficient cell removal, ECM preservation, reduction in chemical residues, and maintenance of biomechanical properties. In particular, the effects of SDC exposure time on these characteristics require further investigation.

Accordingly, this study aimed to optimize a physicochemical porcine tracheal decellularization protocol using SDC by reducing detergent exposure time and evaluating its effects on cell removal in the submucosa and cartilage, ECM preservation, residual SDC, SDC cytotoxicity, cytocompatibility of decellularized matrices, and tissue biomechanical properties. We hypothesized that reducing SDC exposure time would be sufficient to achieve effective submucosal decellularization without requiring complete chondrocyte removal, thereby minimizing ECM alterations and preserving scaffold biomechanical properties.

## 2. Materials and Methods

### 2.1. Experimental Design

This study was designed to optimize a physicochemical porcine tracheal decellularization protocol by evaluating the effects of different sodium deoxycholate (SDC; Sigma-Aldrich, D6750, St. Louis, MO, USA) exposure periods on the removal of cellular components, preservation of the extracellular matrix (ECM), and tissue biomechanical properties.

The experimental design included a Control (In Natura) group and samples subjected to 10 successive decellularization cycles (C1–C10), corresponding to cumulative SDC exposure periods of 24–240 h. Decellularization efficiency across cycles was assessed by residual DNA quantification, hematoxylin and eosin (H&E) histological analysis, and residual SDC quantification. Based on this assessment, C1, C2, and C10, corresponding to 24, 48, and 240 h of SDC exposure, respectively, were selected for additional ECM characterization, biomechanical assessment, and cytocompatibility testing.

The experimental workflow comprised tracheal procurement and preparation, decellularization and washing, assessment of decellularization efficiency, selection of experimental cycles, and subsequent histological and biochemical ECM characterization, biomechanical analysis, and cytocompatibility assessment.

### 2.2. Procurement and Preparation of Porcine Tracheas

The experimental protocol was approved by the Animal Use Ethics Committee of the Faculdade de Medicina da Universidade de São Paulo (CEUA-FMUSP, protocol 1673/2021). One hundred porcine tracheas were obtained from a slaughterhouse immediately after slaughter of 10-month-old Large White pigs weighing approximately 100 kg. After collection, the tracheas were stored on ice and transported to the laboratory within 24 h.

In the laboratory, 63 tracheas were manually prepared to obtain 630 fragments, individually sectioned with a metal knife and standardized to an approximate area of 3.85 cm^2^. Of these, 600 fragments were equally distributed among 6050-mL conical tubes, with 10 fragments per tube, and assigned to the 10 decellularization cycles (C1–C10), with six tubes per cycle (n = 6 experimental units). The remaining 30 fragments were maintained in natura and used as the Control group, being reserved as untreated Control samples for the different analyses. The remaining 37 tracheas were allocated to biomechanical characterization. For this purpose, larger tracheal segments were subjected to the decellularization protocol and subsequently used to prepare longitudinal and transverse specimens for tensile testing. Additional tracheas were allocated to account for potential sample loss during specimen preparation. The final biomechanical analysis included five specimens from five different tracheas per group (Control, C1, C2, and C10; n = 5) for each testing direction.

### 2.3. Decellularization Protocol

Porcine tracheal fragments were decellularized using a physicochemical method with 4% SDC as an ionic detergent to promote cell lysis and removal of cellular components. The protocol was based on a previously established method for tracheal tissue decellularization, with modifications intended to optimize detergent exposure time [16].

**Figure 1:**
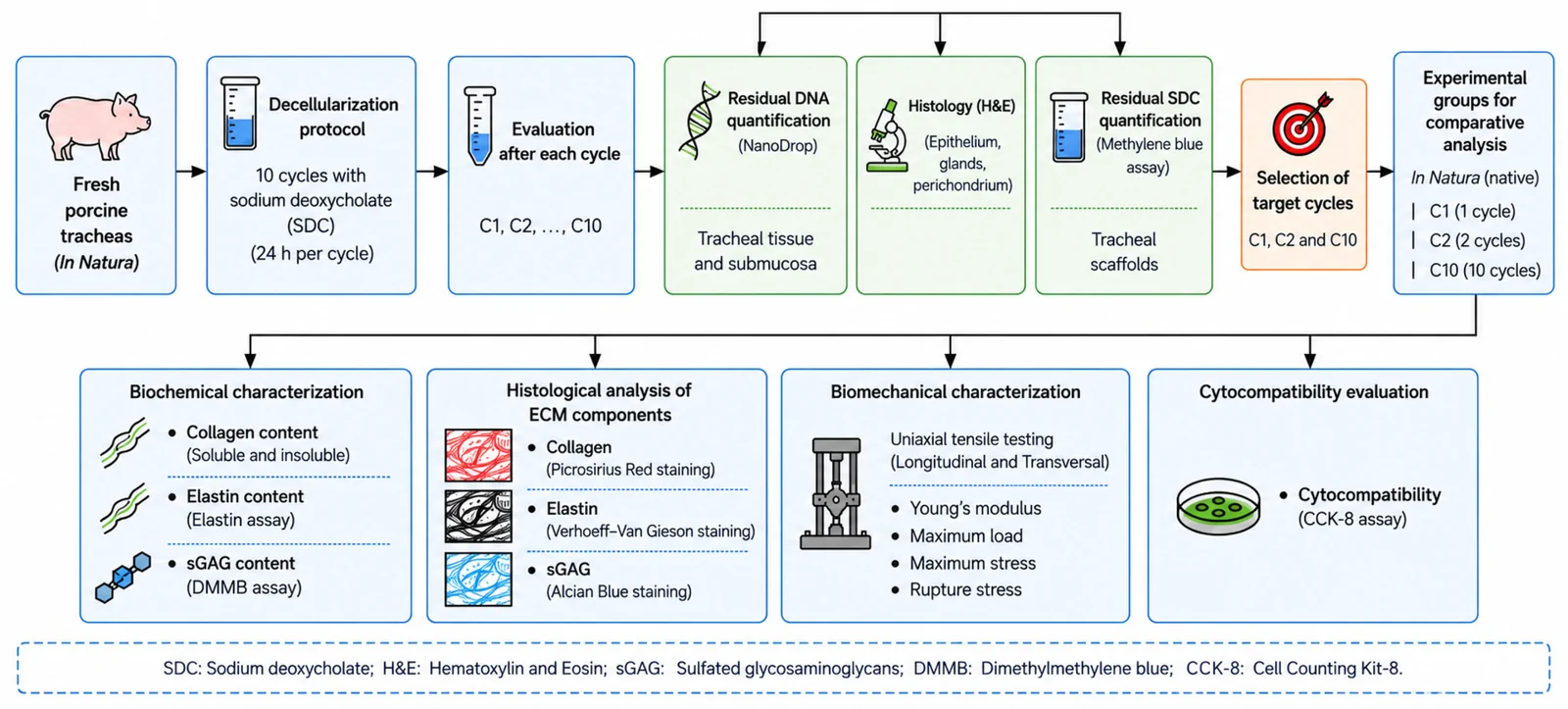
Experimental design for optimization of the decellularization protocol and characterization of porcine tracheas.

The fragments were exposed to 4% SDC under constant agitation at 180 rpm and 37 °C. Each cycle consisted of 24 h of SDC exposure, with up to 10 consecutive cycles (C1–C10), corresponding to cumulative exposure periods of 24–240 h. At each cycle, six tubes containing 10 fragments each were removed from the decellularization process and allocated to subsequent analyses (Figure 1).

Decellularization efficiency was initially assessed across C1–C10 by residual DNA quantification and H&E histological analysis. Based on the findings of this initial assessment, C1, C2, and C10 were selected for subsequent characterization, representing the first decellularization cycle, the immediately subsequent cycle, and the condition with the longest SDC exposure, respectively. Residual SDC was quantified in all decellularization cycles (C1–C10). The selected cycles were subsequently subjected to ECM characterization, cytocompatibility assessment, and biomechanical characterization.

### 2.4. Washing Steps and Enzymatic Treatment

All washing steps were performed at 37 °C under constant agitation at 180 rpm. For fragments assigned to subsequent decellularization cycles, the SDC solution was removed at the end of each cycle, and the fragments underwent three successive washes with 30 mL of deionized water for 10 min each. After washing, fresh 4% SDC solution was added, and the fragments proceeded to the next cycle. This procedure was repeated until each experimental condition reached the predetermined number of cycles.

At the end of the final cycle for each experimental condition (C1–C10), the fragments underwent the final washing protocol. Sequential washes were initially performed with 30 mL of deionized water for 30 min, 30 mL of 0.9% NaCl solution (Sigma-Aldrich, S5886, St. Louis, MO, USA) for 30 min, 30 mL of deionized water for 1 h, and 30 mL of 0.9% NaCl solution for 1 h.

**Figure 2:**
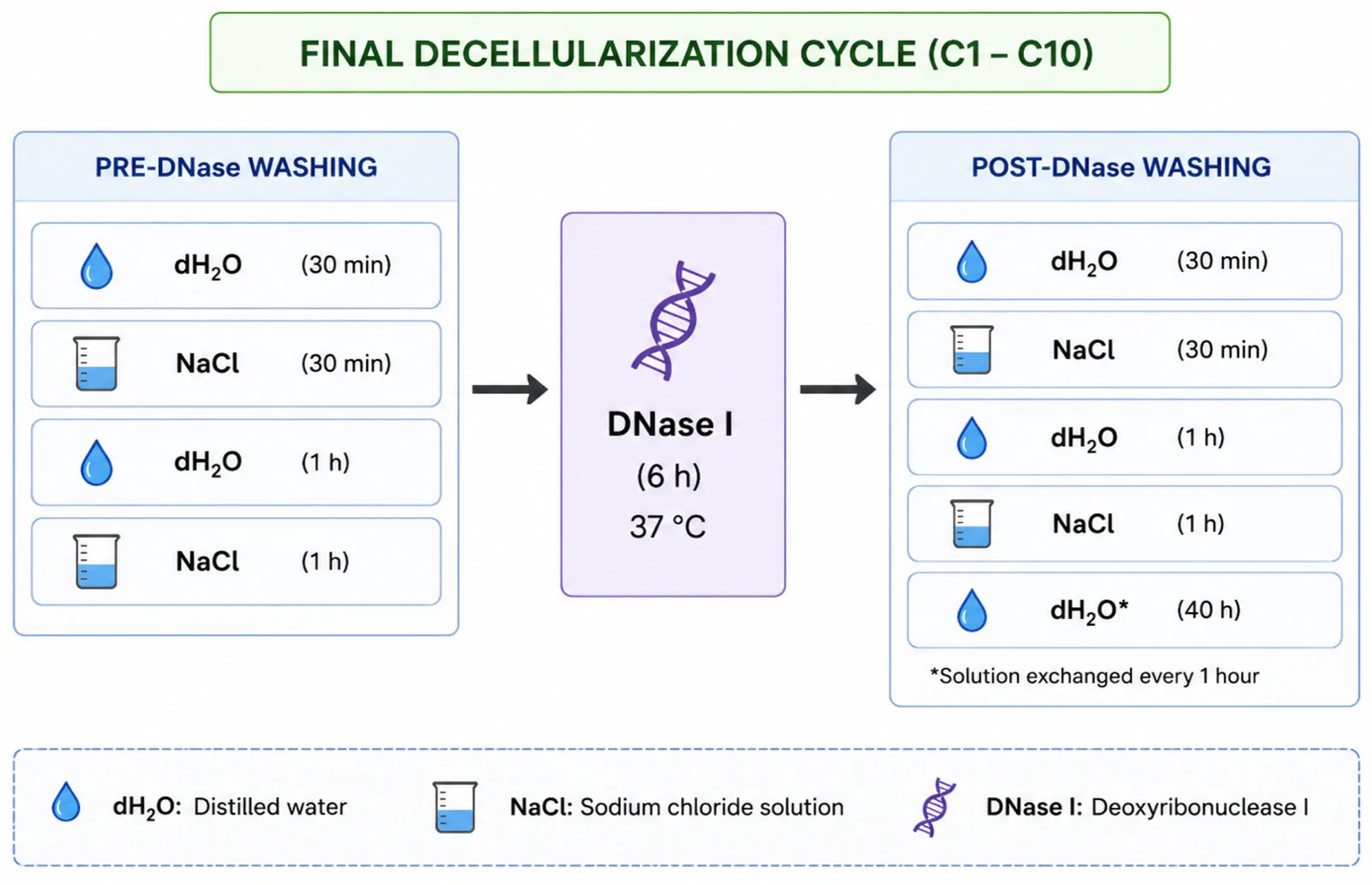
Washing sequence and DNase I treatment after porcine tracheal decellularization cycles.

The fragments were then subjected to enzymatic treatment with DNase I (Roche Diagnostics GmbH, Cat. No. 11 284 932 001, Mannheim, Germany) at 30 µg/mL, prepared in an activation solution containing 1.3 mM MgSO_4_ (M7506, Sigma-Aldrich, St. Louis, MO, USA) and 2 mM CaCl_2_ (C4901, Sigma-Aldrich, St. Louis, MO, USA), for 6 h at 37 °C. After enzymatic treatment, the sequence of washes with deionized water and 0.9% NaCl solution was repeated, maintaining the respective durations of 30 min and 1 h for each solution. Finally, the fragments underwent a prolonged wash with deionized water for 40 h, with water changes every 1 h (Figure 2).

### 2.5. Residual DNA Quantification

For residual DNA assessment, the submucosa was separated from the tracheal fragments, allowing independent analysis of the submucosa and of the remaining tracheal tissue after submucosal removal, hereafter referred to as tracheal tissue. Samples from the Control (In Natura) group and C1–C10 were fragmented, lyophilized, and weighed.

For each sample, 300 mg of lyophilized tissue was incubated overnight at 55 °C in a digestion solution containing 1× extraction buffer [75 mM NaCl and 25 mM ethylenediaminetetraacetic acid (EDTA; EDS, Sigma-Aldrich, St. Louis, MO, USA)], 10% sodium dodecyl sulfate (SDS; L050-07, J.T.Baker, Phillipsburg, NJ, USA), and proteinase K (13-7823-01, Nova Biotecnologia, São Paulo, SP, Brazil). DNA was extracted using 6 M NaCl and chloroform (C1062.01.BJ, Synth, Diadema, SP, Brazil), precipitated with absolute ethanol (1.00983, Merck KGaA, Darmstadt, Germany), washed with ≥70% ethanol, and resuspended in DNase- and RNase-free water (W4502, Sigma-Aldrich, St. Louis, MO, USA).

DNA concentration was determined by spectrophotometry using the NanoDrop One (Thermo Fisher Scientific, Waltham, MA, USA), and results were expressed as ng DNA/mg dry tissue. A value of 50 ng/mg dry tissue was used as a reference parameter for characterization of decellularized matrices and was not considered, in isolation, a criterion of decellularization efficiency.

### 2.6. Histological Assessment

Representative samples from the Control (In Natura) group and C1–C10 were fixed, processed, and embedded for histological assessment. Hematoxylin and eosin (H&E) staining was performed for all cycles to qualitatively assess removal of cellular components and preservation of tissue architecture. In C1, C2, and C10, Picrosirius Red, Alcian Blue, and Verhoeff–Van Gieson staining was additionally performed to assess collagen, glycosaminoglycans, and elastic fibers, respectively. Slides were evaluated by light microscopy for qualitative analysis of the presence, distribution, and organization of tissue components.

### 2.7. Residual SDC Quantification

Residual sodium deoxycholate (SDC) content was determined in the Control (In Natura) group and decellularization cycles C1–C10 using a colorimetric method based on complex formation between SDC and methylene blue, as described by Mathapati et al. and modified by Zvarova et al. [17,18].

Tracheal fragments underwent overnight enzymatic digestion at 55 °C using 1 mL of 1× extraction buffer (75 mM NaCl and 25 mM EDTA) and 80 µL of proteinase K. After digestion, 80 µL of homogenate was added to 420 µL of 0.0125% methylene blue solution, followed by 1000 µL of chloroform, for a final volume of 1.5 mL. After phase separation, 100 µL of the lower phase was transferred to a 96-well plate, and absorbance was measured at 630 nm using SpectraMax i3 microplate reader (Molecular Devices, San Jose, CA, USA).

Residual SDC concentration was determined from a standard curve containing SDC concentrations ranging from 0.0625% to 4%, and results were expressed as the percentage of residual SDC relative to tissue dry mass (*w*/*w*).

### 2.8. Glycosaminoglycan Quantification

Glycosaminoglycan (GAG) content was determined in samples from the Control (In Natura) group and C1, C2, and C10 using a colorimetric method based on the methodology described by Farndale et al. [19]. After sample digestion, GAGs were quantified by colorimetric reaction, and absorbance was measured at 525 nm using SpectraMax i3 microplate reader (Molecular Devices). Results were normalized to the mass of tissue analyzed.

### 2.9. Collagen Quantification

Soluble and insoluble collagen contents were determined in samples from the Control (In Natura) group and C1, C2, and C10. Soluble collagen was quantified using the Sircol™ 2.0 Soluble Collagen Assay (SIRC2, BioColor Ltd., Carrickfergus, UK), whereas insoluble collagen was quantified using the Sircol™ 2.0 Insoluble Collagen Assay (BioColor Ltd., Carrickfergus, UK), according to the manufacturer’s instructions. Absorbance was measured at 556 nm using a SpectraMax i3 microplate reader (Molecular Devices). Results were normalized to the mass of tissue analyzed.

### 2.10. Elastin Quantification

Elastin content was determined in samples from the Control (In Natura) group and C1, C2, and C10 using the Fastin™ Elastin Assay Kit (F2000/F4000, BioColor Ltd., Carrickfergus, UK) according to the manufacturer’s instructions. Absorbance was measured at 513 nm using a SpectraMax i3 microplate reader (Molecular Devices), and the results were normalized to the mass of tissue analyzed.

### 2.11. Determination of the SDC Cytotoxicity Threshold

Immortalized human tracheobronchial epithelial BEAS-2B cells (95102433, Culture Collections, UK Health Security Agency, UK) at passage P6 were cultured in LHC-9 medium (12680013, Gibco, Life Technologies Corporation, Grand Island, NY, USA) and seeded in 12.5-cm^2^ culture flasks at a density of 1 × 10^5^ cells per flask.

To determine the cytotoxicity threshold of sodium deoxycholate (SDC), cells were exposed to concentrations of 0.0002%, 0.0004%, 0.0009%, 0.0019%, 0.0039%, 0.0078%, 0.0156%, 0.0312%, 0.0625%, 0.125%, 0.25%, 0.5%, 1%, 2%, and 4%, in addition to the Control (0% SDC), for 24, 48, and 72 h. After each exposure period, viable cells were identified by Trypan Blue exclusion (0.4%; T8154, Sigma-Aldrich, St. Louis, MO, USA) and counted using a Neubauer chamber. The number of viable cells at each SDC concentration was normalized to the Control, which was considered 100% viability. Cell viability ≥70% relative to the Control was considered non-cytotoxic, whereas values <70% were considered indicative of cytotoxicity. SDC concentrations corresponding to 70% viability were determined by interpolation of concentration–response curves fitted by nonlinear regression [20,21].

### 2.12. Cytocompatibility Assessment of Decellularized Matrices

The cytocompatibility of decellularized tracheal matrices was assessed using an indirect extraction method according to previously described methodologies [19,20]. Lyophilized tracheal fragments were incubated in LHC-9 medium at a ratio of 100 mg/mL for 24 h to obtain extracts. After incubation, the extracts were collected and filtered through 0.22-µm syringe filters before cell exposure.

BEAS-2B cells were seeded in 96-well plates (1 × 10^4^ cells/well) and, after attachment, exposed to extracts at concentrations of 25%, 50%, 75%, and 100% for 24, 48, and 72 h. Cell viability was assessed using the Cell Counting Kit-8 (CCK-8; 96992, Sigma-Aldrich, St. Louis, MO, USA), and absorbance was measured at 450 nm using SpectraMax i3 microplate reader (Molecular Devices). Results were expressed as a percentage relative to the Control, consisting of cells maintained in culture medium without exposure to extracts. Cell viability ≥70% relative to the Control was considered non-cytotoxic, whereas values <70% were considered indicative of cytotoxicity [22].

### 2.13. Biomechanical Characterization

For biomechanical characterization, the tracheal segments were prepared from specimens for tensile strength testing, as we described previously by our group [16]. In short, tracheas sectioned longitudinally along the midline of the membranous portion were laid flat. Specimens obtained from the anterior tracheal wall in the longitudinal and transverse orientations and prepared in a dog-bone configuration, size V, according to ASTM D638-02a [23].

Prior to tensile testing, specimens underwent three rapid preconditioning cycles automatically performed by the testing system. Uniaxial tensile tests used an Instron 3365 universal testing machine and Bluehill 3 software (Instron, Norwood, MA, USA) at a speed of 10 mm/min. During testing, samples were immersed in saline solution at 36 °C to prevent dehydration and maintain near-physiological conditions.

Because specimen thickness was irregular due to the alternation between cartilage and connective tissue, cross-sectional area was determined from the mass and density of each sample to estimate volume and subsequently calculating the area. Stress–strain curves were recorded by the software, and the biomechanical parameters analyzed included Young’s modulus (MPa), maximum load (N), maximum stress (MPa), and stress at break (MPa). Analyses were performed separately for the longitudinal and transverse orientations.

### 2.14. Artificial Intelligence-Assisted Figure Preparation

Artificial intelligence ChatGPT (GPT-5.6 Sol, OpenAI) was used solely for the graphical preparation of Figure 1 and Figure 2 and the Graphical Abstract. All scientific information presented in these figures was verified by the authors against the original experimental data and results. AI was used exclusively for graphical layout and did not generate, modify, or affect the scientific content or experimental results.

### 2.15. Statistical Analysis

Data were expressed as mean ± standard deviation (SD). For residual DNA analysis comparing tracheal tissue and submucosa across decellularization cycles, two-way analysis of variance (two-way ANOVA) was used, with tissue type and decellularization cycle (C1–C10) as factors. For the remaining between-group comparisons, one-way analysis of variance (one-way ANOVA) was used. When applicable, analyses were followed by Bonferroni multiple-comparison tests.

## 3. Results

### 3.1. Optimization of the Decellularization Protocol

The efficiency of the decellularization protocol was initially assessed over 10 successive cycles of exposure to 4% SDC. Process characterization included residual DNA quantification, histological analysis by hematoxylin and eosin (H&E) staining, and residual SDC quantification across the different decellularization cycles. Together, these analyses characterized removal of cellular and nuclear components, changes in tissue organization, and the presence of detergent residues after processing.

#### 3.1.1. Residual DNA Quantification

**Figure 3:**
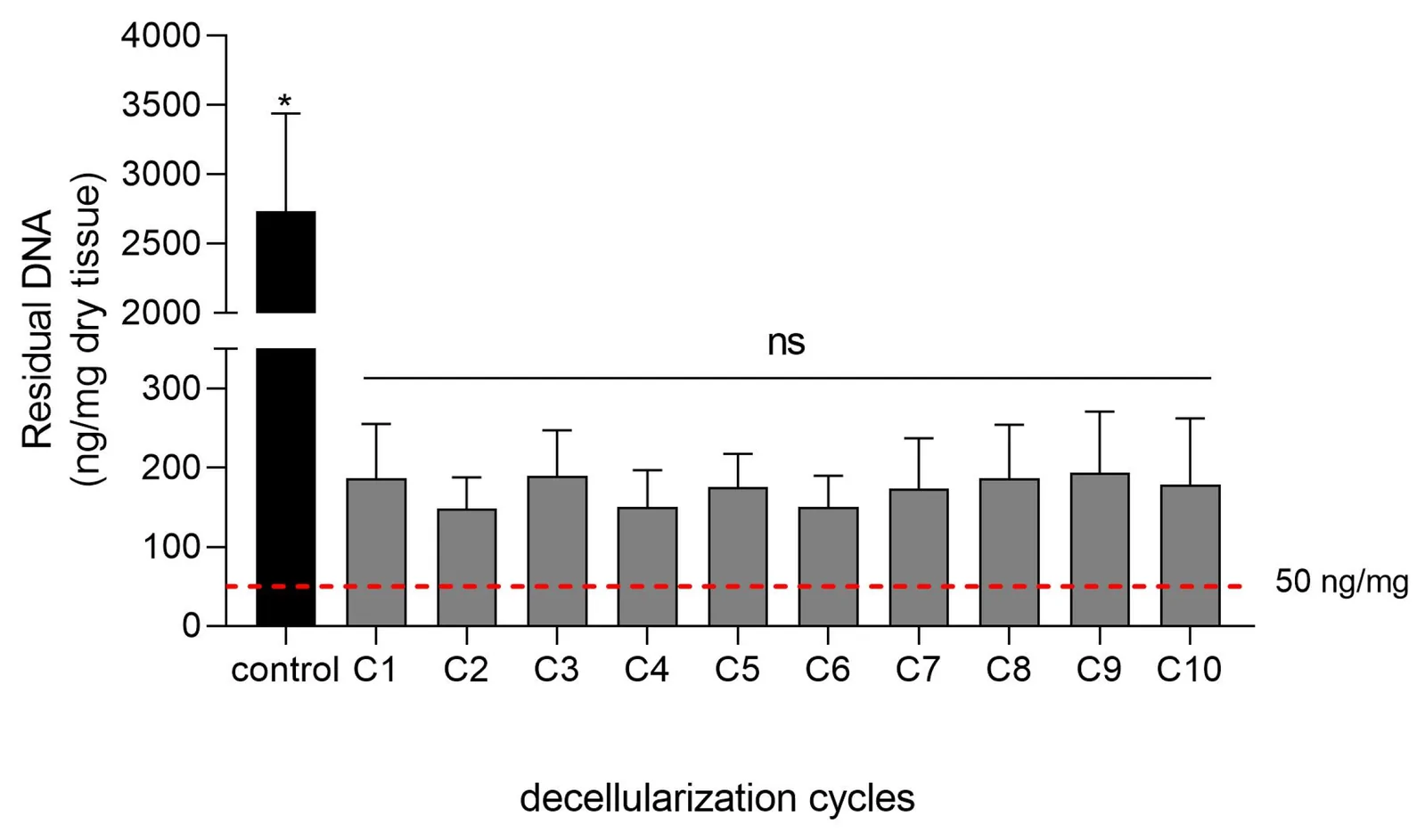
Quantification of residual DNA (ng/mg dry tissue) in tracheal tissue from the Control group and after different decellularization cycles (C1–C10). Bars represent mean values ± standard deviation (n = 4–6 per group). The dashed line indicates the reference threshold of 50 ng/mg dry tissue. Overall differences among groups were assessed by one-way ANOVA (*p* < 0.001), followed by Bonferroni’s multiple-comparisons test. The asterisk (*) indicates that all decellularization cycles were significantly different from the Control group (adjusted *p* < 0.001 for all comparisons). ns indicates no statistically significant differences among the decellularization cycles (adjusted *p* ≥ 0.05).

In tracheal fragments, significant overall differences among groups were observed (ANOVA, *p* < 0.001). Decellularization reduced DNA content by 93.7% relative to the Control group, with significantly lower values in all decellularization cycles compared with the Control (Bonferroni-adjusted *p* < 0.001 for all comparisons). No significant differences were observed among C1–C10. However, values remained above 50 ng/mg dry tissue in all cycles evaluated (Figure 3).

**Figure 4:**
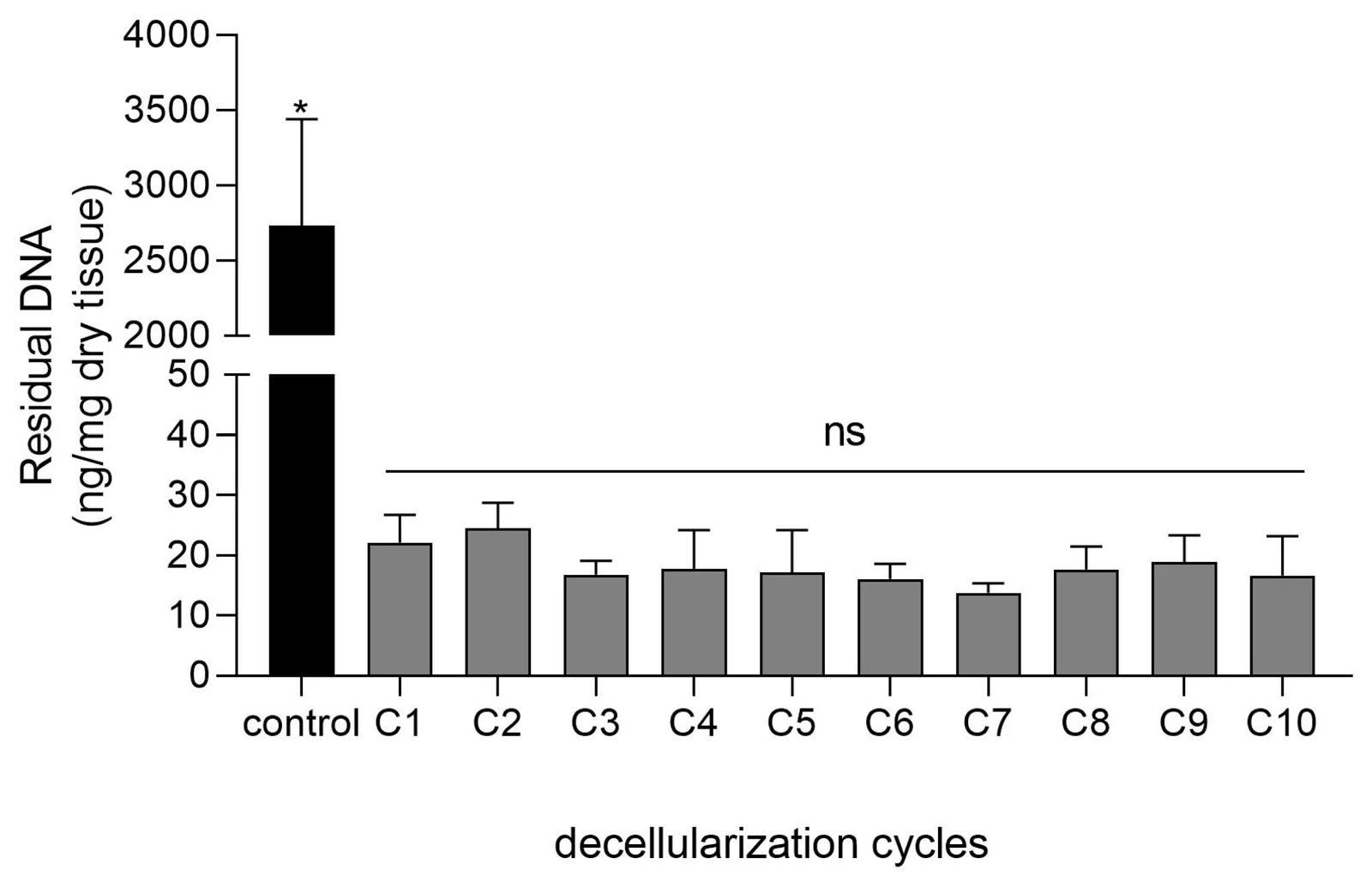
Quantification of residual DNA (ng/mg dry tissue) in the tracheal submucosa from the Control group and after different decellularization cycles (C1–C10). Bars represent mean values ± standard deviation (n = 4–5 per group). The *Y*-axis contains a scale break to improve visualization of the differences between the Control group and the decellularization cycles. Overall differences among groups were assessed by one-way ANOVA (*p* < 0.001), followed by Bonferroni’s multiple-comparisons test. The asterisk (*) indicates that all decellularization cycles were significantly different from the Control group (adjusted *p* < 0.001 for all comparisons). ns indicates no statistically significant differences among the decellularization cycles (adjusted *p* ≥ 0.05).

**Figure 5:**
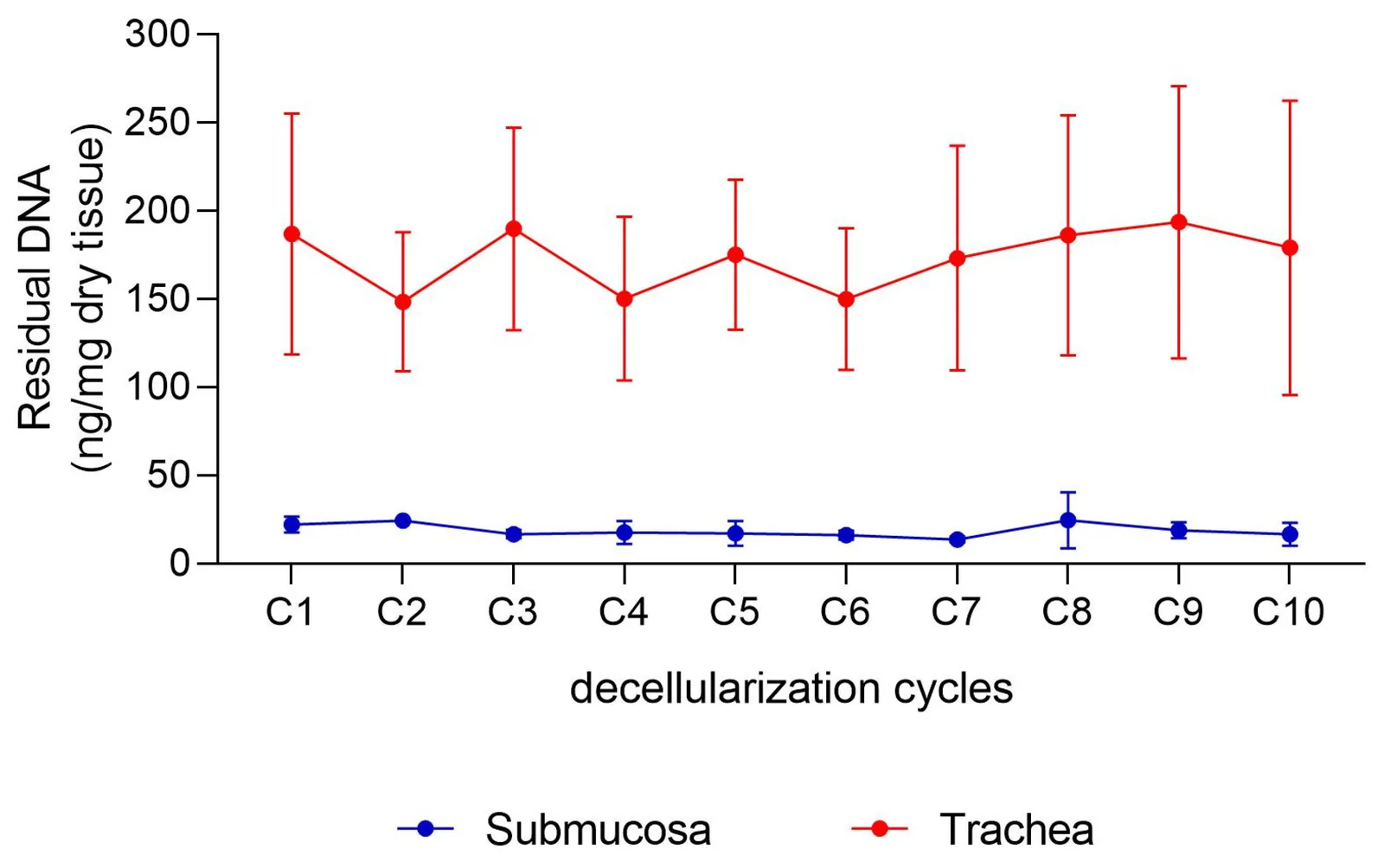
Quantification of residual DNA (ng/mg dry tissue) in tracheal tissue and the submucosa after different decellularization cycles (C1–C10). Points represent mean values, and error bars represent standard deviation (n = 3–6 per group). Data were analyzed by ordinary two-way ANOVA, which showed a significant effect of tissue type (*p* < 0.001), with no significant effect of decellularization cycle (*p* = 0.946) and no significant tissue type × decellularization cycle interaction (*p* = 0.946), followed by Bonferroni’s multiple-comparisons test. Residual DNA content was significantly lower in the submucosa than in tracheal tissue at each decellularization cycle (adjusted *p* < 0.001 for all comparisons).

In the submucosa, significant overall differences among groups were observed (ANOVA, *p* < 0.001). DNA content was reduced by 99.3% relative to the Control, with significantly lower values in all decellularization cycles compared with the Control (Bonferroni-adjusted *p* < 0.001 for all comparisons). No significant differences were observed among C1–C10 (Bonferroni-adjusted *p* ≥ 0.05). Values were below 50 ng/mg dry tissue from the first cycle onward and remained below this threshold in subsequent cycles (Figure 4). Residual DNA concentration in the submucosa was significantly lower than that in tracheal tissue across the decellularization cycles (two-way ANOVA, main effect of tissue type, *p* < 0.001), with no significant effect of decellularization cycle (*p* = 0.946) or tissue type × cycle interaction (*p* = 0.946). Bonferroni-adjusted multiple comparisons confirmed significantly lower residual DNA levels in the submucosa than in tracheal tissue at all decellularization cycles (adjusted *p* < 0.001 for all comparisons; (Figure 5)).

#### 3.1.2. Histological Analysis by H&E

**Figure 6:**
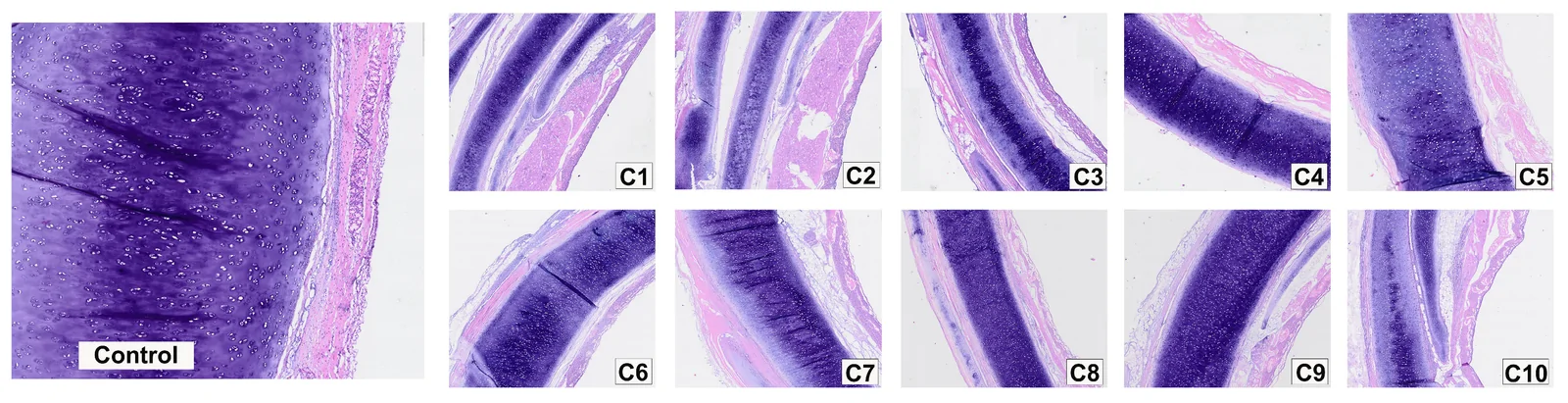
Histological evaluation of porcine tracheas subjected to decellularization using hematoxylin and eosin (H&E) staining. Representative photomicrographs of the Control group and tracheal fragments subjected to different decellularization cycles (C1–C10), showing a reduction in cellular components in the submucosa, persistence of chondrocytes within the cartilage, and preservation of the overall tissue architecture. Magnification: 40×; field of view: 1964 × 1744 µm (width × height) for all panels.

Histological analysis demonstrated a marked reduction in cellular components in the submucosa as early as the first decellularization cycle (C1), corresponding to 24 h of SDC exposure, compared with in natura tissue. Subsequent cycles showed a similar histological pattern, with no evident differences in cell removal among C1, C2, and C10. In contrast, chondrocytes remained present in the cartilage after decellularization, while the overall tissue architecture remained preserved across the cycles evaluated (Figure 6).

#### 3.1.3. Residual Sodium Deoxycholate Quantification

**Figure 7:**
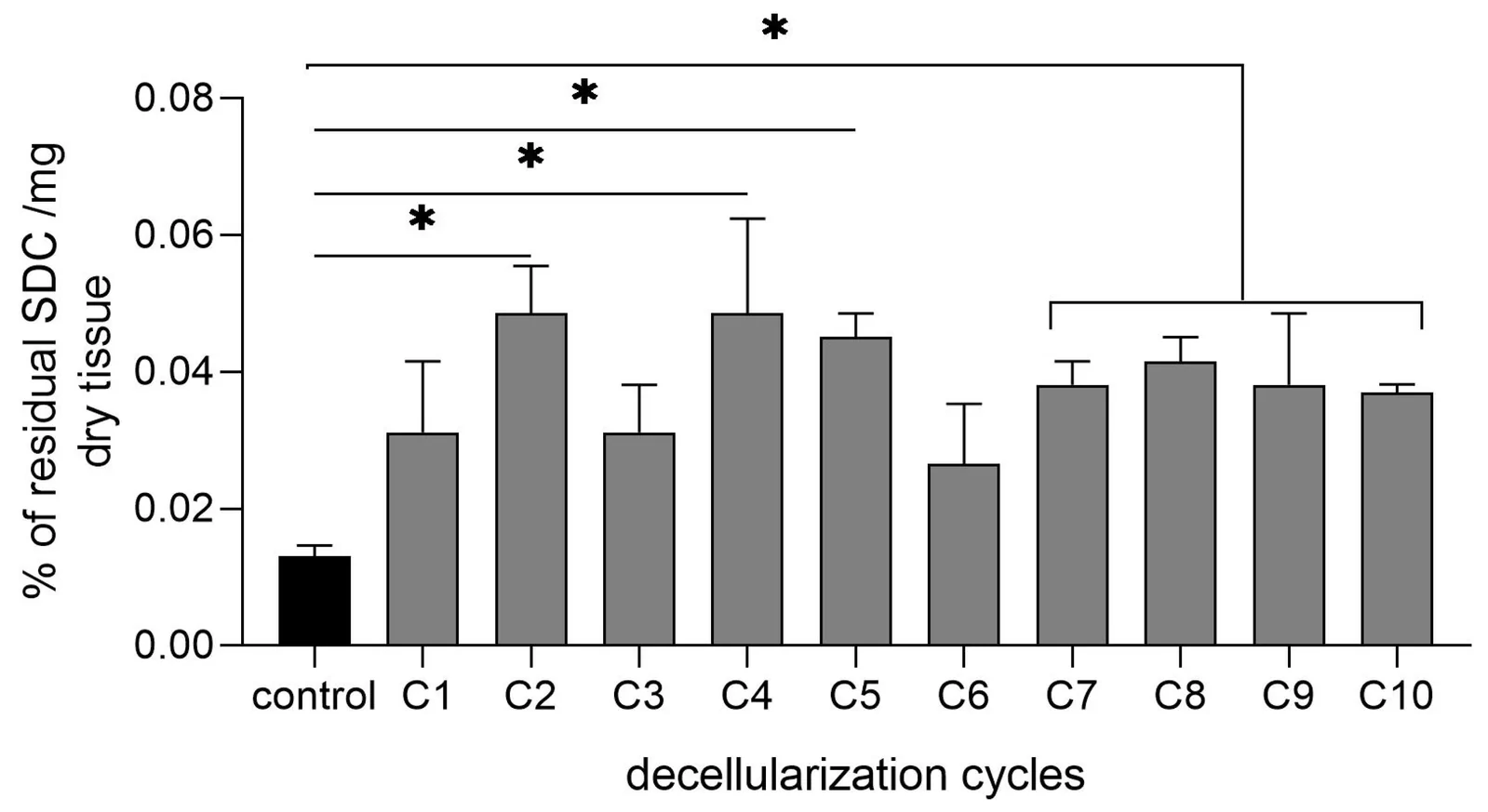
Quantification of residual SDC in samples after different decellularization cycles. Bars represent mean values ± standard deviation (n = 3 per group). Control: *in natura* tissue; C1–C10: samples subjected to the respective decellularization cycles. Overall differences among groups were assessed by one-way ANOVA (*p* < 0.001), followed by Bonferroni’s multiple-comparisons test comparing the Control with each decellularization cycle. The asterisk (*) indicates a statistically significant difference compared with the Control (adjusted *p* < 0.05). Significant differences were observed for C2, C4, and C5 (adjusted *p* < 0.001), C7 and C9 (adjusted *p* = 0.005), C8 (adjusted *p* = 0.001), and C10 (adjusted *p* = 0.008). No significant differences were observed for C1 and C3 (adjusted *p* = 0.074) or C6 (adjusted *p* = 0.387).

Residual SDC quantification, expressed relative to tissue dry mass (*w*/*w*), showed significant overall differences among groups (ANOVA, *p* < 0.001). Bonferroni-adjusted multiple comparisons showed significantly higher values in C2, C4, C5, C7, C8, C9, and C10 compared with the Control (adjusted *p* < 0.05), whereas C1, C3, and C6 showed no significant differences (adjusted *p* ≥ 0.05; Figure 7).

### 3.2. Quantification of Extracellular Matrix Components

**Figure 8:**
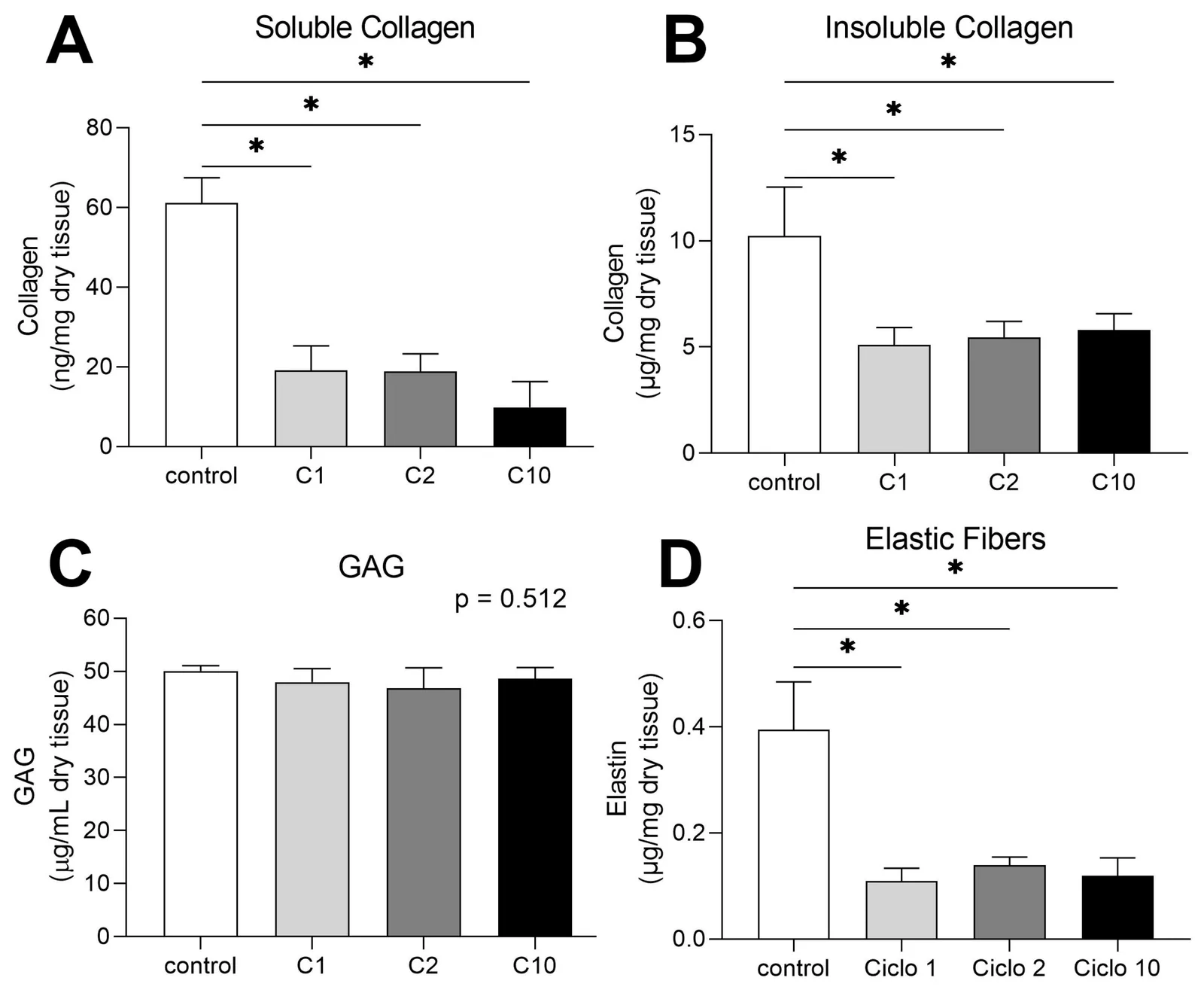
Quantification of extracellular matrix components in porcine tracheal fragments subjected to decellularization. (**A**) Soluble collagen; (**B**) insoluble collagen; (**C**) glycosaminoglycans (GAGs); and (**D**) elastic fibers in tissues from the Control group and decellularization cycles 1 (C1), 2 (C2), and 10 (C10). Results are expressed as mean ± standard deviation. Sample sizes were n = 6 per group for soluble collagen, insoluble collagen, and elastic fibers, and n = 3–4 per group for GAGs. Overall differences among groups were assessed by one-way ANOVA (soluble collagen, *p* < 0.001; insoluble collagen, *p* < 0.001; GAGs, *p* = 0.512; elastic fibers, *p* < 0.001). Multiple comparisons between the Control group and each decellularization cycle were performed using Bonferroni’s multiple-comparisons test. Horizontal lines indicate statistically significant comparisons with the Control; * indicates an adjusted *p* < 0.05.

ECM composition was assessed by quantifying soluble collagen, insoluble collagen, GAGs, and elastin in the Control group and C1, C2, and C10. Significant overall differences among groups were observed for soluble and insoluble collagen (ANOVA, *p* < 0.001). Bonferroni-adjusted multiple comparisons showed significantly lower soluble and insoluble collagen contents in C1, C2, and C10 than in the Control (adjusted *p* < 0.001 for all comparisons; Figure 8A,B). Compared with the Control, soluble collagen was reduced by 68.6%, 69.2%, and 84.0% in C1, C2, and C10, respectively, whereas insoluble collagen was reduced by 50.0%, 46.7%, and 43.4%, respectively. GAG quantification showed no significant overall differences among groups (ANOVA, *p* = 0.512; Figure 8C). Elastin content also differed significantly among groups (ANOVA, *p* < 0.001), with significantly lower values in C1, C2, and C10 than in the Control in Bonferroni-adjusted comparisons (adjusted *p* < 0.001 for all comparisons; Figure 8D). Compared with the Control, elastin content was reduced by 72.3%, 64.8%, and 69.7% in C1, C2, and C10, respectively.

### 3.3. Histological Characterization of the Extracellular Matrix

**Figure 9:**
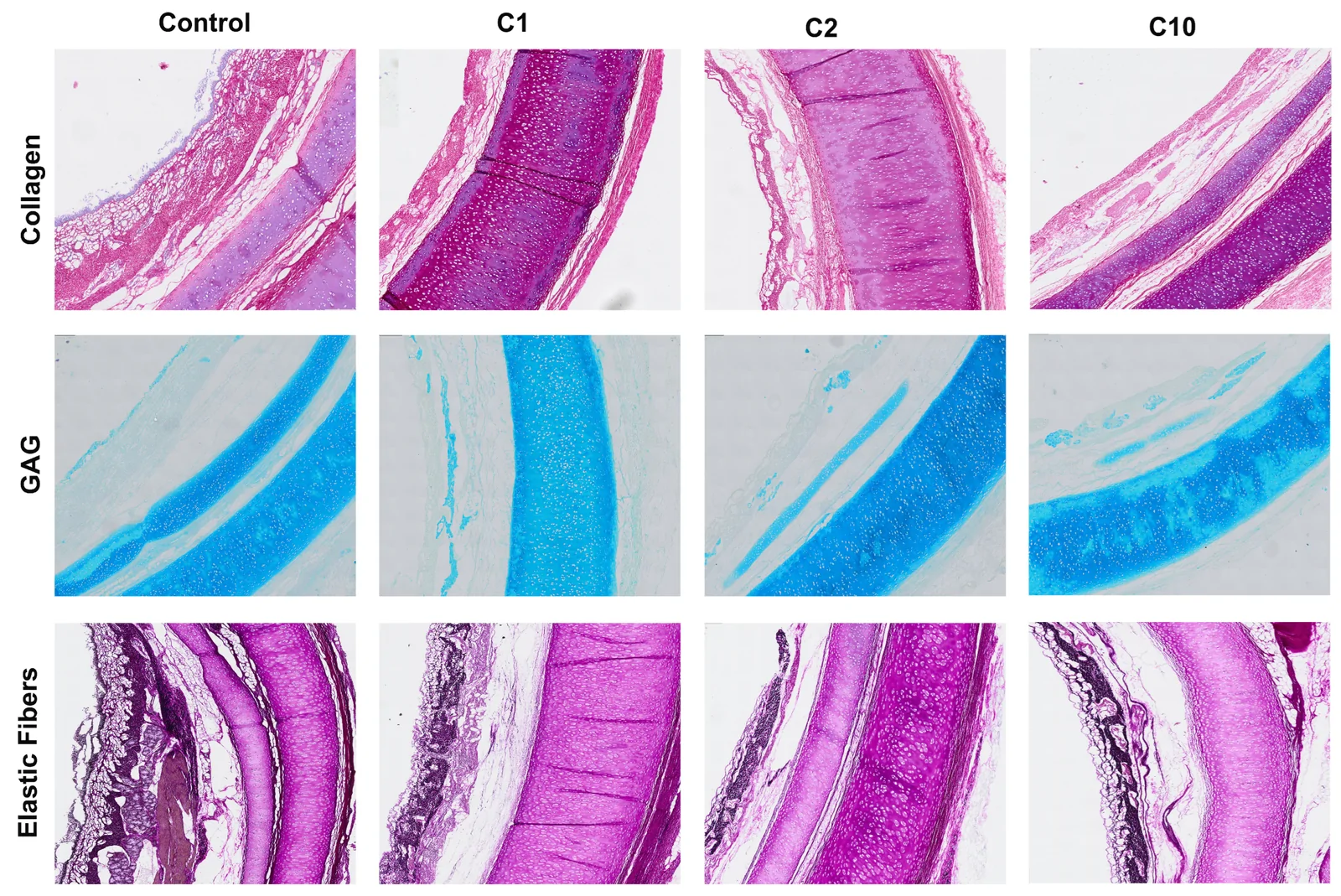
Histological analysis of the main extracellular matrix components of porcine tracheas after different decellularization cycles. Picrosirius Red staining was used to evaluate collagen fibers in the Control group and cycles C1, C2, and C10; Alcian Blue staining was used to visualize glycosaminoglycans (GAGs); and Verhoeff–Van Gieson staining was used to evaluate elastic fibers under the same experimental conditions. Images represent qualitative assessments of the presence, distribution, and organization of extracellular matrix components. Magnification: 40×; field of view: 1964 × 1744 µm (width × height) for all panels.

Histological characterization of the ECM was performed using Picrosirius Red, Alcian Blue, and Verhoeff–Van Gieson staining for qualitative assessment of collagen, GAGs, and elastic fibers, respectively. Picrosirius Red staining demonstrated preservation of collagen fiber organization in C1, C2, and C10, although qualitative differences in staining intensity were observed relative to the Control. Alcian Blue demonstrated preservation of GAG presence and distribution, with a visually similar pattern between the Control and the cycles evaluated. Verhoeff–Van Gieson staining demonstrated preservation of elastic fiber organization despite qualitative differences in staining intensity after decellularization (Figure 9).

### 3.4. Biomechanical Properties

**Figure 10:**
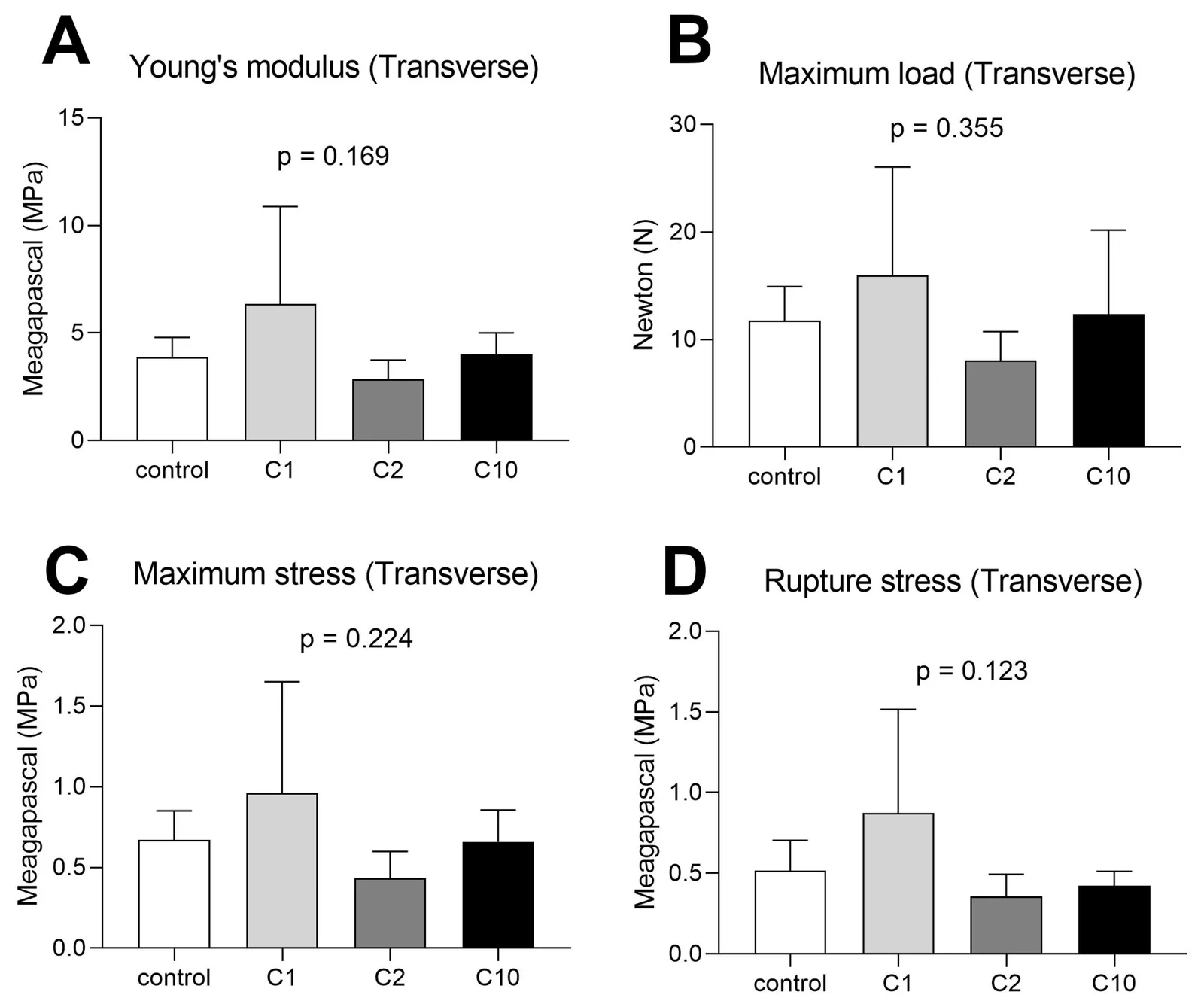
Evaluation of the biomechanical properties of porcine tracheas subjected to the decellularization protocol in the transverse direction. (**A**) Young’s modulus; (**B**) maximum load; (**C**) maximum stress; and (**D**) rupture stress. Control: *in natura* tissue; C1, C2, and C10: samples subjected to the respective decellularization cycles. Data are presented as mean ± standard deviation (n = 5 per group). Overall differences among groups were assessed by one-way ANOVA, with the corresponding *p* value indicated in each panel. No significant overall differences among groups were observed for Young’s modulus (*p* = 0.169), maximum load (*p* = 0.355), maximum stress (*p* = 0.224), or rupture stress (*p* = 0.123).

In the transverse direction, no significant overall differences among groups were observed for Young’s modulus (ANOVA, *p* = 0.169), maximum load (ANOVA, *p* = 0.355), maximum stress (ANOVA, *p* = 0.224), or rupture stress (ANOVA, *p* = 0.123; Figure 10A–D).

**Figure 11:**
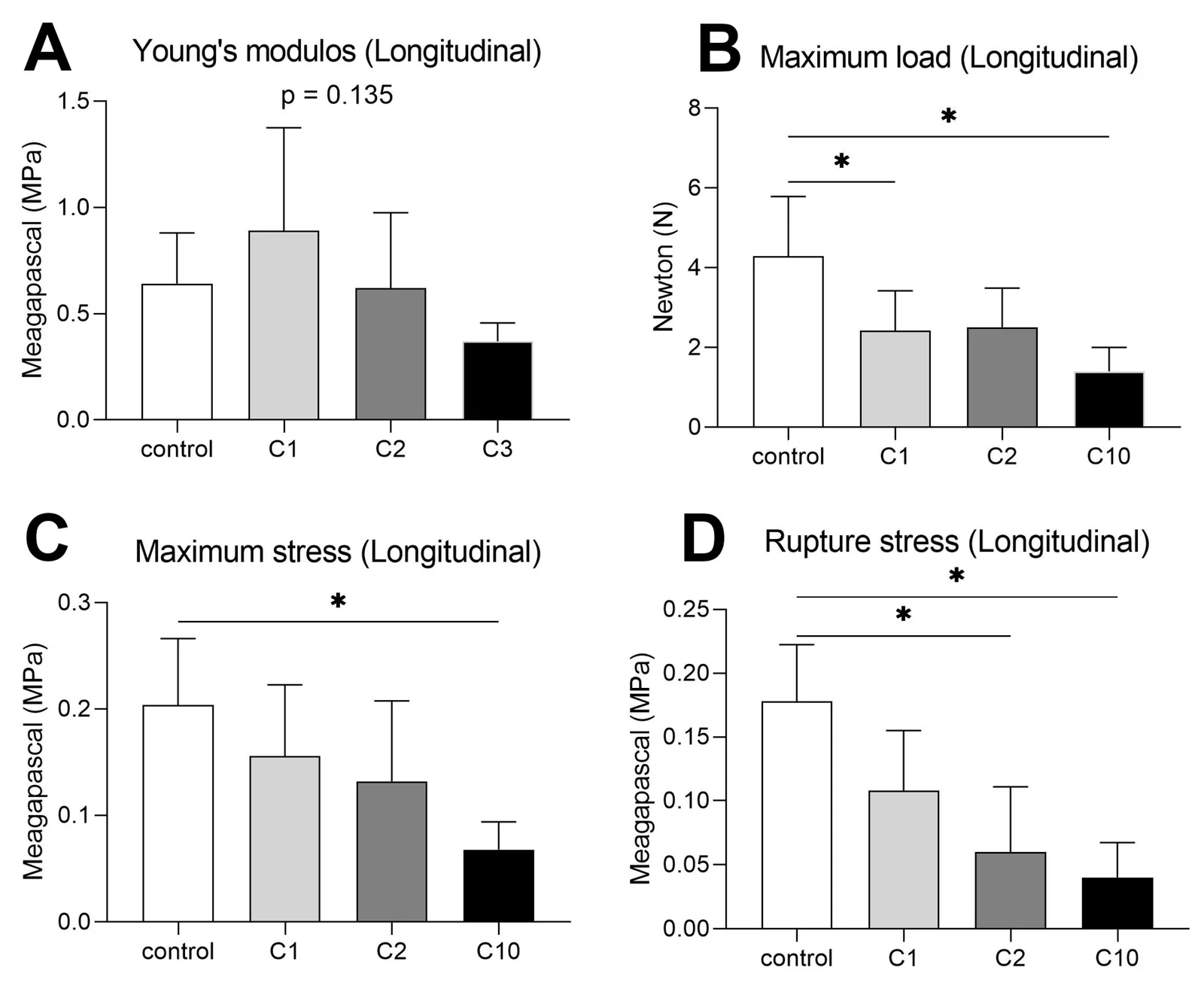
Evaluation of the biomechanical properties of porcine tracheas subjected to the decellularization protocol in the longitudinal direction. (**A**) Young’s modulus; (**B**) maximum load; (**C**) maximum stress; and (**D**) rupture stress. Control: *in natura* tissue; C1, C2, and C10: samples subjected to the respective decellularization cycles. Data are presented as mean ± standard deviation (n = 5 per group). Overall differences among groups were assessed by one-way ANOVA (Young’s modulus, *p* = 0.135; maximum load, *p* = 0.005; maximum stress, *p* = 0.020; rupture stress, *p* < 0.001). Multiple comparisons between the Control group and each decellularization cycle were performed using Bonferroni’s multiple-comparisons test. Horizontal lines indicate statistically significant comparisons with the Control; * indicates an adjusted *p* < 0.05.

In the longitudinal direction, no significant overall differences among groups were observed for Young’s modulus (ANOVA, *p* = 0.135). However, significant overall differences among groups were observed for maximum load (ANOVA, *p* = 0.005), maximum stress (ANOVA, *p* = 0.020), and rupture stress (ANOVA, *p* < 0.001). Bonferroni-adjusted multiple comparisons showed that maximum load was significantly lower in C1 (adjusted *p* = 0.040) and C10 (adjusted *p* = 0.002), maximum stress was significantly lower in C10 (adjusted *p* = 0.008), and rupture stress was significantly lower in C2 (adjusted *p* = 0.002) and C10 (adjusted *p* < 0.001) compared with the Control (Figure 11A–D).

### 3.5. Determination of the SDC Cytotoxicity Threshold

**Figure 12:**
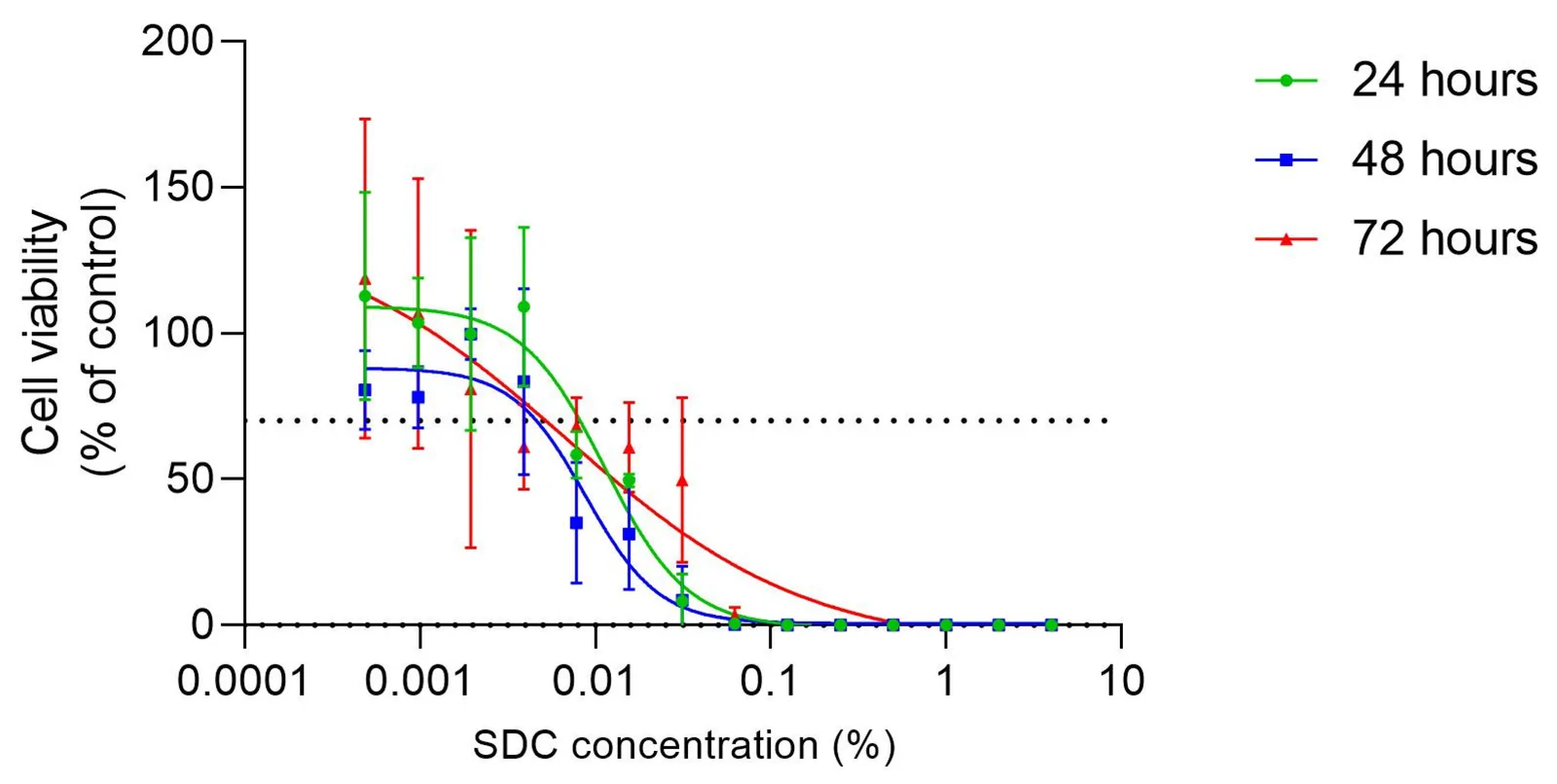
Determination of the SDC cytotoxicity threshold in immortalized human bronchial epithelial cells (BEAS-2B). Cell viability is expressed as a percentage relative to Control. Points represent mean values, and error bars represent standard deviation (n = 3 per concentration and time point). Lines represent concentration–response curves obtained by nonlinear regression for 24, 48, and 72 h. The horizontal dashed line indicates the 70% cell viability threshold adopted as the criterion for cytotoxicity. SDC concentrations are shown on a logarithmic scale.

Assessment of BEAS-2B cell viability demonstrated a concentration-dependent cytotoxic effect of SDC after 24, 48, and 72 h of exposure. Nonlinear regression analysis of the concentration–response curves estimated SDC concentrations corresponding to ≥70% cell viability of 0.008% after 24 h and 0.005% after 48 and 72 h (Figure 12).

### 3.6. Cytocompatibility of Decellularized Matrices

**Figure 13:**
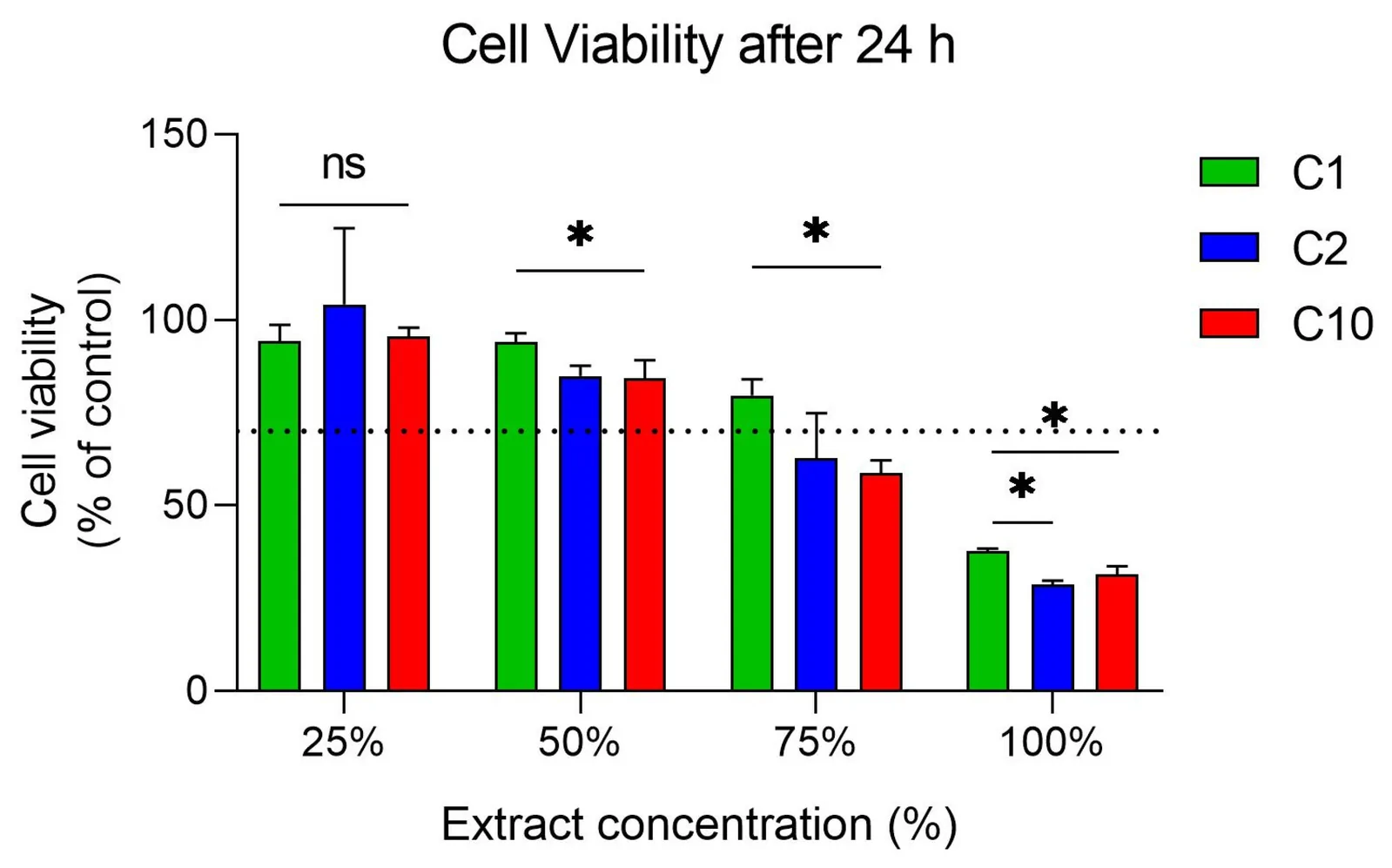
Viability of BEAS-2B cells after 24 h of exposure to extracts from decellularized tracheas. BEAS-2B cells were exposed for 24 h to extracts obtained from tracheas subjected to decellularization cycles C1, C2, and C10 at concentrations of 25%, 50%, 75%, and 100%. The dashed line represents the 70% cell viability threshold adopted as the criterion for cytocompatibility. Data are presented as mean ± standard deviation (n = 3 per group at each extract concentration). Overall differences among decellularization cycles were assessed separately at each extract concentration using one-way ANOVA (25%, *p* = 0.586; 50%, *p* = 0.024; 75%, *p* = 0.035; 100%, *p* = 0.001), followed by Bonferroni’s multiple-comparisons test. Horizontal lines indicate pairwise comparisons; * indicates an adjusted *p* < 0.05, whereas ns indicates no statistically significant difference (adjusted *p* ≥ 0.05).

**Figure 14:**
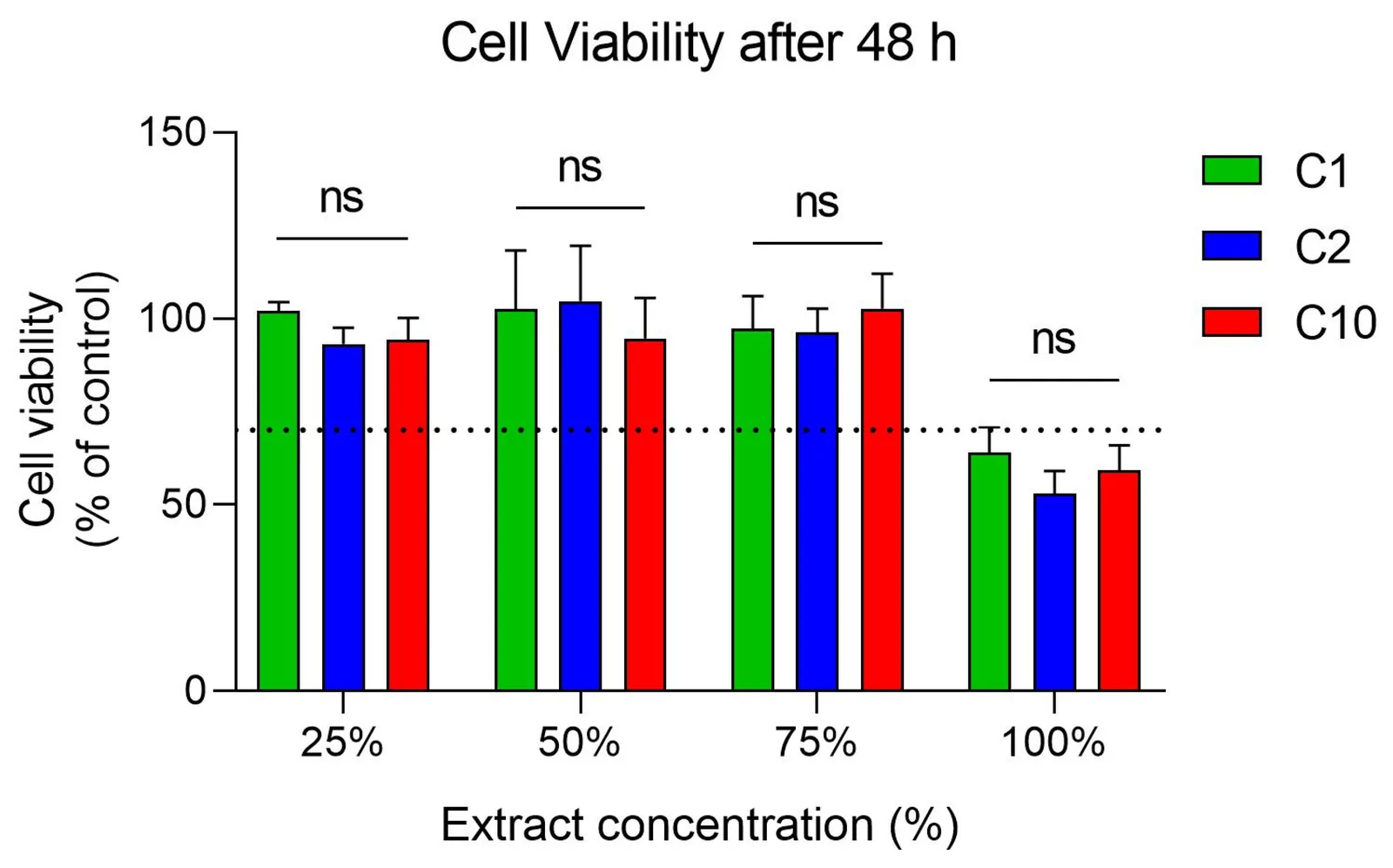
Viability of BEAS-2B cells after 48 h of exposure to extracts from decellularized tracheas. BEAS-2B cells were exposed for 48 h to extracts obtained from tracheas subjected to decellularization cycles C1, C2, and C10 at concentrations of 25%, 50%, 75%, and 100%. The dashed line represents the 70% cell viability threshold adopted as the criterion for cytocompatibility. Data are presented as mean ± standard deviation (n = 3 per group at each extract concentration). Overall differences among decellularization cycles were assessed separately at each extract concentration using one-way ANOVA (25%, *p* = 0.095; 50%, *p* = 0.667; 75%, *p* = 0.618; 100%, *p* = 0.194), followed by Bonferroni’s multiple-comparisons test. Horizontal lines indicate pairwise comparisons; ns indicates no statistically significant difference (adjusted *p* ≥ 0.05).

**Figure 15:**
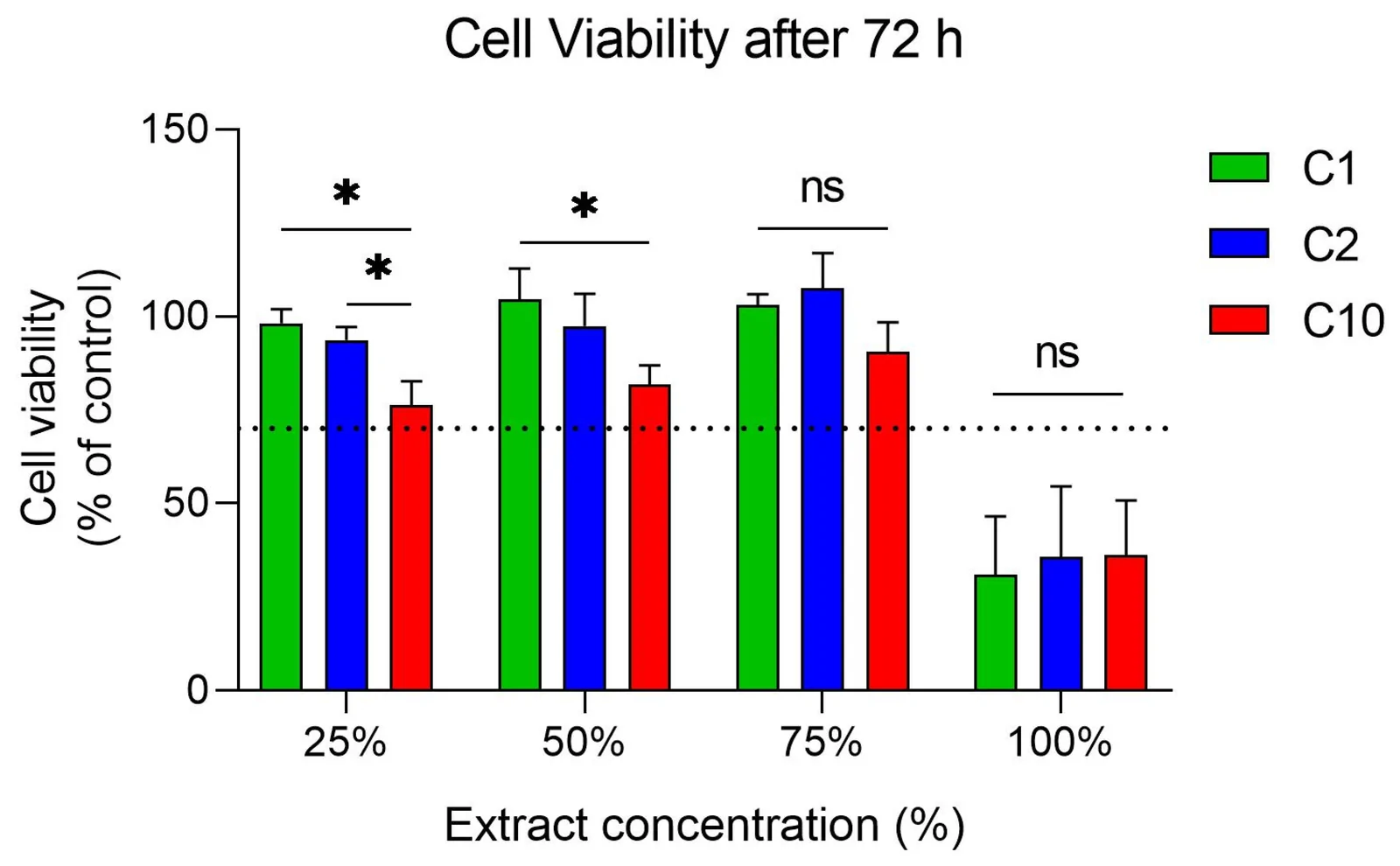
Viability of BEAS-2B cells after 72 h of exposure to extracts from decellularized tracheas. BEAS-2B cells were exposed for 72 h to extracts obtained from tracheas subjected to decellularization cycles C1, C2, and C10 at concentrations of 25%, 50%, 75%, and 100%. The dashed line represents the 70% cell viability threshold adopted as the criterion for cytocompatibility. Data are presented as mean ± standard deviation (n = 3 per group at each extract concentration). Overall differences among decellularization cycles were assessed separately at each extract concentration using one-way ANOVA (25%, *p* = 0.003; 50%, *p* = 0.026; 75%, *p* = 0.066; 100%, *p* = 0.916), followed by Bonferroni’s multiple-comparisons test. Horizontal lines indicate pairwise comparisons; * indicates an adjusted *p* < 0.05, whereas ns indicates no statistically significant difference (adjusted *p* ≥ 0.05).

The cytocompatibility of decellularized matrices was assessed based on the viability of BEAS-2B cells exposed to extracts from C1, C2, and C10 at concentrations of 25%, 50%, 75%, and 100% after 24, 48, and 72 h. Overall, diluted extracts showed cell viability above the 70% threshold, although differences among cycles were observed at certain concentrations and exposure times. In contrast, undiluted extracts (100%) showed viability below 70% for all three cycles after 24, 48, and 72 h (Figure 13, Figure 14 and Figure 15).

After 24 h, no significant overall differences among cycles were observed at 25% extract concentration (ANOVA, *p* = 0.586). Significant overall differences among cycles were observed at 50% (ANOVA, *p* = 0.024), 75% (ANOVA, *p* = 0.035), and 100% (ANOVA, *p* = 0.001). Bonferroni-adjusted multiple comparisons showed significant differences between C1 and C10 at 50% (adjusted *p* = 0.042) and 75% (adjusted *p* = 0.049). At 100%, significant differences were observed between C1 and C2 (adjusted *p* = 0.001) and between C1 and C10 (adjusted *p* = 0.006; Figure 13). After 48 h, no significant overall differences among decellularization cycles were observed at 25% (ANOVA, *p* = 0.095), 50% (ANOVA, *p* = 0.667), 75% (ANOVA, *p* = 0.618), or 100% (ANOVA, *p* = 0.194) extract concentrations (Figure 14). After 72 h, significant overall differences among decellularization cycles were observed at 25% (ANOVA, *p* = 0.003) and 50% (ANOVA, *p* = 0.026) extract concentrations. Bonferroni-adjusted multiple comparisons showed significant differences between C1 and C10 (adjusted *p* = 0.004) and between C2 and C10 (adjusted *p* = 0.012) at 25%, and between C1 and C10 at 50% (adjusted *p* = 0.030). No significant overall differences among cycles were observed at 75% (ANOVA, *p* = 0.066) or 100% (ANOVA, *p* = 0.916; Figure 15).

## 4. Discussion

This study demonstrates that optimization of the physicochemical porcine tracheal decellularization protocol substantially reduced SDC exposure time, with effective submucosal decellularization achieved in the first processing cycle. DNA quantification and histological analysis showed a reduction in cellular components in the submucosa, whereas chondrocytes remained present in the cartilage. Increasing the number of cycles provided no relevant additional benefit to decellularization. These findings are particularly important because prolonged detergent exposure increases processing time and may promote undesirable ECM alterations. Previous studies have emphasized the importance of optimizing decellularization conditions to balance removal of cellular components with structural preservation of the scaffold [1,4,21,22].

The protocol used in this study employed 4% SDC, a concentration previously described in tracheal decellularization protocols [23]. The 4% SDC disrupts cell membrane and solubilizes the intracellular components. It can also affect the ECM components, particularly during prolonged exposure conditions [2,6,24]. In our study, increasing the number of decellularization cycles did not benefit the submucosal decellularization, and the chondrocytes remained present within the cartilaginous matrix. These findings suggest that prolonging the exposure to 4% SDC to increase cell removal may be unnecessary when adequate submucosal decellularization has been already achieved after the first cycle.

Reducing the 4% SDC exposure time has practical and methodological relevance. In this study, the first cycle, corresponding to 24 h of detergent exposure, was sufficient to achieve effective submucosal decellularization despite the persistence of chondrocytes in the cartilage. Limiting detergent exposure may help reduce potential ECM alterations, considering that longer or more aggressive treatments can compromise structural matrix components [2,6,24]. These results reinforce the importance of establishing the shortest exposure time required to achieve the objectives of decellularization while avoiding prolonged treatments that provide no additional benefit.

Quantitative analysis of residual DNA revealed an important feature. In the tracheal tissue remaining after submucosal removal, no statistically significant differences in DNA content were observed among decellularization cycles, and values remained above the reference criterion of 50 ng DNA/mg dry tissue proposed for characterization of decellularized tissues. However, this result should be interpreted cautiously, because this threshold alone does not constitute a universal criterion for determining decellularization success. Assessment of the process should consider, in addition to residual DNA quantity, its distribution within the tissue, the presence of nuclear material, ECM preservation, and functional scaffold characteristics [2,6,24].

Tracheal cartilage has structural characteristics that may hinder decellularization. Its dense extracellular matrix, rich in collagen and proteoglycans, may limit the diffusion of decellularizing agents and hinder the removal of cells located in deeper tissue regions. In addition, the location of chondrocytes within lacunae represents an additional challenge to cartilage decellularization [8,12,24].

In this context, partial decellularization has been proposed as an alternative strategy aimed at removing epithelial and submucosal cellular components while preserving the underlying cartilage and its resident chondrocytes. Bergman et al. [25] demonstrated that partially decellularized tracheal grafts maintained gross tracheal architecture, with removal of epithelial and submucosal structures and preservation of cartilage-associated collagen and GAGs. Importantly, donor-derived chondrocytes remained detectable in the grafts for up to 6 months after orthotopic implantation, although their number was reduced at 6 months compared with preimplantation and syngeneic controls. The authors suggested that preservation of viable chondrocytes may be relevant to cartilage homeostasis, while emphasizing that the functional significance of the observed changes in chondrocyte number remains unclear.

In our study, the separate analysis of tracheal tissue and submucosa also provided relevant information. In the submucosa, residual DNA values were below 50 ng/mg dry tissue from the first cycle onward and remained below this value in subsequent cycles. In contrast, the tracheal tissue remaining after submucosal removal showed values above this threshold. Comparative analysis between tracheal tissue and submucosa demonstrated lower residual DNA content in the submucosa in all cycles evaluated. Moreover, the absence of a significant effect of cycle number and of interaction between tissue type and cycle indicates that prolonged SDC exposure did not produce any additional detectable reduction in residual DNA in either tissue. This finding suggests that the higher residual DNA content observed in tracheal tissue may be related, at least in part, to the persistence of chondrocytes in the cartilage. Thus, combined analysis of residual DNA in tracheal tissue and submucosa allows a more appropriate interpretation of decellularization efficiency in the tracheal wall.

ECM characterization demonstrated preservation of glycosaminoglycan (GAG) content, with no statistically significant differences between the Control group and the decellularization cycles evaluated. This finding is relevant because GAGs play important roles in ECM organization and hydration, contribute to tissue viscoelastic properties, and participate in interactions with growth factors and other bioactive molecules [2,26]. Although ionic detergents may affect GAG content depending on decellularization conditions [3,6,24,27,28], increasing the number of cycles did not result in a detectable progressive loss of these components under the experimental conditions evaluated. These results indicate that GAGs were relatively preserved during decellularization despite the use of SDC.

In contrast, substantial reductions in collagen and elastin were observed after decellularization. Compared with the Control group, soluble collagen was reduced by 68.6%, 69.2%, and 84.0% in C1, C2, and C10, respectively; insoluble collagen by 50.0%, 46.7%, and 43.4%; and elastin by 72.3%, 64.8%, and 69.7%, respectively. These findings demonstrate that relevant changes in ECM composition were already present during the initial decellularization cycles, although the magnitude of these alterations did not show a uniform pattern of increase with the number of cycles across all ECM components evaluated.

Alterations in ECM components have also been reported in detergent-based tracheal decellularization protocols. In porcine tracheas subjected to repeated decellularization cycles using 4% SDC, Partington et al. reported mean values corresponding to reductions of approximately 75%, 77%, and 88% in soluble collagen at cycles 10, 20, and 25, respectively, relative to the corresponding time-matched controls, whereas total collagen content was not significantly altered [28]. Differences among studies should, however, be interpreted in the context of detergent concentration, duration and conditions of exposure, and the specific ECM fraction evaluated.

The configuration of the samples may also have contributed to the magnitude of the ECM losses observed in the present study. Because decellularization was performed using tracheal fragments, the greater surface-area-to-tissue-volume ratio, together with the presence of cut surfaces directly accessible to the decellularization solution, may have facilitated SDC penetration and interaction with the ECM. As the effect of sample geometry was not directly evaluated, this possibility should be regarded as a hypothesis.

Preservation of the ECM is relevant not only to the biomechanical behavior of the scaffold but also to subsequent cell–matrix interactions. Previous studies have associated changes in ECM composition after decellularization with alterations in scaffold mechanical properties [28,29]. In addition to their structural roles, ECM components participate in cell adhesion, migration, and differentiation, and preservation of proteins such as collagen, fibronectin, and laminin may therefore be relevant to subsequent scaffold repopulation [7,28]. Thus, the reductions in collagen and elastin observed in the present study may have implications for future recellularization; however, because cell adhesion, migration, and repopulation were not directly evaluated, their functional consequences remain to be determined. Likewise, although recellularization and subsequent tissue remodeling may result in the deposition of new ECM [30], it cannot be assumed that these processes will fully restore the composition, organization, or biomechanical properties of native tissue.

In this context, partial decellularization or targeted de-epithelialization strategies may represent an alternative approach to limit ECM exposure to decellularizing agents [31]. A double-chamber bioreactor was used to deepithelialize porcine tracheal segments by luminal perfusion with 1% SDS for 3 h, while the outer chamber was perfused with culture medium to support chondrocyte viability. This approach preserved most viable chondrocytes and ECM components, including collagen, elastin, GAGs, type IV collagen, laminin, and type II collagen, without significant alterations in the mechanical properties evaluated. The de-epithelialized grafts also supported BEAS-2B cell attachment and viability and maintained viable epithelial cells on the luminal surface following repopulation in the bioreactor. Considering that removal of epithelial and submucosal cellular components was achieved after the first cycle in the present study, future approaches based on shorter and more spatially controlled luminal exposure may represent a strategy to limit detergent exposure of the deeper layers of the tracheal wall and potentially favor ECM preservation.

Histological findings complemented the quantitative ECM component data, demonstrating preservation of the overall organization of collagen, glycosaminoglycans, and elastic fibers after decellularization. Because histological analyses were based on representative slides, these findings should be interpreted qualitatively. Nevertheless, histological assessment, together with quantitative characterization of ECM components, provides complementary information on the effects of decellularization on matrix composition and organization.

The biomechanical assessment provided an additional functional perspective on the effects of decellularization. The trachea exhibits complex, anisotropic, and nonlinear mechanical behavior, with distinct responses depending on tissue orientation. Eskandari et al. [32] demonstrated this anisotropic behavior in porcine airways, emphasizing the importance of considering tissue orientation when interpreting tensile mechanical properties. In a previous study using decellularized porcine tracheas, longitudinal specimens comprised cartilage and dense connective tissue between the cartilaginous rings, whereas transverse specimens consisted predominantly of a single cartilaginous ring, indicating that mechanical properties measured along these two axes reflect distinct structural characteristics of the tracheal wall [16].

In the transverse axis, no statistically significant differences were found between the Control group and the cycles evaluated for any of the biomechanical parameters analyzed. This suggests that decellularization did not produce detectable changes in transverse mechanical behavior under the experimental conditions studied. This finding is particularly relevant given the contribution of cartilaginous ring organization and ECM architecture to circumferential strength and maintenance of airway patency [33,34].

In contrast, changes in biomechanical parameters were observed in the longitudinal axis. Although Young’s modulus did not differ significantly among groups, reductions in maximum load, maximum stress, and rupture stress were observed in specific decellularization cycles. C1 showed reduced maximum load, C2 showed reduced rupture stress, and C10 showed changes in maximum load, maximum stress, and rupture stress. Thus, alterations were more extensive in C10. These results suggest that prolonged SDC exposure may contribute to changes in certain longitudinal mechanical properties of the tissue, although some alterations were already observed in the initial cycles. Exposure to decellularizing agents may affect ECM integrity and organization and consequently modify scaffold biomechanical behavior [28,29,33].

The reductions in longitudinal mechanical parameters occurred concomitantly with the reductions in collagen and elastin content observed in the present study. However, because ECM fiber organization, alignment, and integrity were not directly evaluated, a direct mechanistic relationship between the biochemical and biomechanical alterations cannot be established. This distinction is important because the mechanical properties of the trachea depend not only on the amount of individual ECM components but also on their structural organization and orientation, which contribute to the anisotropic behavior of the tissue [32].

These findings indicate that the decellularization protocol is associated with changes in scaffold composition and biomechanical properties. The clinical significance of the reduced longitudinal mechanical strength remains uncertain, particularly because recellularization and subsequent tissue remodeling may further modify ECM composition and scaffold biomechanical properties [30]. Therefore, we cannot accurately anticipate the potential impact on anastomotic integrity and respiratory mechanics, which remain speculative and require further functional evaluation.

Moreover, the present uniaxial tensile tests characterize tissue-level mechanical properties but do not reproduce the integrated behavior of an intact tubular trachea under physiological conditions. Ex vivo testing does not fully account for factors such as tissue prestress, boundary conditions imposed by surrounding tissues, and multidirectional loading. Complementary approaches, such as tubular inflation or biaxial testing, may more adequately reproduce the complexity of physiological airway loading [32].

Another relevant finding of this study was the presence of residual SDC after processing. Detergent quantification showed that efficient cell removal does not necessarily imply complete elimination of the detergent from the scaffold. Detergent residues may remain associated with the ECM after decellularization and compromise cell viability and behavior in subsequent steps [6,7,24]. This issue is particularly relevant for scaffolds intended for recellularization, in which residual cytotoxic agents may limit biological application.

Determination of the SDC cytotoxicity threshold in immortalized human tracheobronchial epithelial cells (BEAS-2B) demonstrated a progressive reduction in cell viability with increasing detergent concentration. By interpolation of the fitted curves, SDC concentrations corresponding to 70% cell viability were estimated at 0.008% after 24 h and 0.005% after 48 and 72 h. These results demonstrate that even low SDC concentrations can compromise cell viability and reinforce the importance of adequate removal of residual detergent from scaffolds before recellularization.

Assessment of cell viability in extracts from decellularized tracheas provided complementary information on scaffold cytocompatibility. Overall, diluted extracts showed cell viability above the 70% threshold, although some variations were observed among cycles and exposure times. In contrast, undiluted extracts (100%) showed viability below 70% in all cycles evaluated after 24, 48, and 72 h. These results indicate lower cytocompatibility of undiluted extracts and reinforce that decellularization efficiency alone is insufficient to ensure scaffold biological compatibility.

The washing step is also important for protocol optimization because adequate removal of residual detergent is required for scaffolds intended for cellular applications [6,24]. In this context, residual SDC quantification, together with determination of its cytotoxicity threshold and assessment of extract cytocompatibility, provided complementary characterization of the chemical and biological effects of the decellularization protocol.

Taken together, the results demonstrate that increasing the number of decellularization cycles provided no relevant additional benefit in terms of cell removal, whereas alterations in ECM components and longitudinal biomechanical parameters were observed with processing. In contrast, 24 h of SDC exposure was sufficient to achieve effective submucosal decellularization, with chondrocytes remaining in the cartilage and preservation of relevant structural and biomechanical characteristics. These findings support the first cycle as the most favorable condition among those evaluated, although the presence of residual SDC and lower cytocompatibility of undiluted extracts highlight the need to optimize the washing steps.

One of the main contributions of this study was to demonstrate that optimization of a decellularization protocol should not be based exclusively on maximizing cell removal. Defining an optimized condition requires integration of different parameters, including residual DNA, preservation of ECM components, biomechanical properties, residual detergent, and cytocompatibility. This approach is particularly important for complex tissues such as the trachea, in which decellularization efficiency must be balanced against preservation of ECM architecture and functional properties [2,3,35].

Several limitations should be considered. Histological assessment was qualitative and based on representative slides, and quantitative histomorphometry analysis was not performed. In addition, although separate analysis of the submucosa and tracheal tissue allowed better interpretation of residual DNA, the immunological consequences of retained chondrocytes were not directly evaluated in this study. Specifically, MHC-II expression and cytokine profiles were not assessed; therefore, no conclusions regarding the immunological acceptability of residual chondrocytes can be drawn from our findings. Future studies may complement these findings through quantitative analyses of ECM organization, recellularization studies, and functional evaluation of the scaffolds.

Overall, the findings demonstrate that reducing SDC exposure time is a viable strategy for obtaining tracheal scaffolds, allowing effective submucosal decellularization while retaining chondrocytes in the cartilage. The 24-h condition was the most favorable among those evaluated when cell removal, preservation of relevant ECM characteristics, and biomechanical properties were considered together. However, detection of residual SDC and lower cytocompatibility of undiluted extracts indicate that the washing steps should be further improved before future recellularization applications.

## 5. Conclusions

Optimization of the porcine tracheal decellularization protocol demonstrated that a single 24-h cycle of SDC exposure was sufficient to substantially reduce cellular components. The protocol preserved structural ECM characteristics and relevant biomechanical properties, particularly in the transverse axis, although alterations in certain longitudinal mechanical parameters were observed after decellularization and were more extensive in the cycle with the longest exposure time.

The presence of residual SDC in the matrices highlighted the importance of adequate detergent removal for cytocompatibility. Determination of the cytotoxicity threshold in BEAS-2B cells, together with assessment of decellularized tracheal extracts, demonstrated that SDC residues can compromise cell viability, underscoring the importance of the washing step in the protocol.

Taken together, the results demonstrate that the quality of a decellularized tracheal matrix should be assessed not only by cell removal but also by ECM and biomechanical property preservation, residual detergent removal, and cytocompatibility. The condition based on 24 h of SDC exposure was the most favorable among those evaluated for obtaining decellularized tracheal matrices; however, the washing still requires optimization before future recellularization applications and functional assessment.

## Data Availability

The data presented in this study are available on request from the corresponding author.

## Abbreviations

The following abbreviations are used in this manuscript:

ANOVA	Analysis of variance
CCK-8	Cell Counting Kit-8
DNA	Deoxyribonucleic acid
ECM	Extracellular matrix
EDTA	Ethylenediaminetetraacetic acid
GAGs	Glycosaminoglycans
H&E	Hematoxylin and eosin
SDC	Sodium deoxycholate
